# Magnesium Deficiency Accelerates Gut Aging and Increases Susceptibility to Colitis

**DOI:** 10.1111/acel.70446

**Published:** 2026-03-16

**Authors:** Rou Zhang, Meiling Ge, Meng Hu, Yanjie Zhao, Baochen Chong, Wanmeng Li, Jia Yu, Ying Lu, Siyu He, Jiao Wang, Jirong Yue, Hai‐Ning Chen, Heng Xu, Yong Peng, Peng Lei, Zuyun Liu, Lunzhi Dai

**Affiliations:** ^1^ National Clinical Research Center for Geriatrics, State Key Laboratory of Biotherapy, West China Hospital Sichuan University Chengdu China; ^2^ Department of Big Data in Health Science School of Public Health, Zhejiang Key Laboratory of Intelligent Preventive Medicine, Center for Clinical Big Data and Analytics Second Affiliated Hospital Zhejiang University School of Medicine Zhejiang Hangzhou China; ^3^ Advanced Mass Spectrometry Center, Research Core Facility, Frontiers Science Center for Disease‐Related Molecular Network, West China Hospital Sichuan University Chengdu China; ^4^ Department of General Surgery, Colorectal Cancer Center, West China Hospital Sichuan University Chengdu China

## Abstract

Aging is linked to a higher incidence of gut diseases such as inflammatory bowel disease (IBD), yet the underlying mechanisms remain unclear. We identified an age‐related decline in magnesium (Mg) levels specifically in the gut across species, prompting investigation of its role in intestinal health. Functional studies demonstrated that Mg restriction accelerates gut aging in old but not in young mice and aggravates colitis severity. Multi‐omics analysis of mouse tissues revealed that dietary Mg deficiency reshapes the phosphoproteome and *N*‐glycoproteome, destabilizing adhesion complexes, a hallmark of intestinal aging and inflammation. In the UK Biobank cohort (*n* = 182,213), dietary Mg intake was inversely correlated with gut disorder risk, with 334.7–420.0 mg/day conferring significant protection against Crohn's disease, ulcerative colitis, irritable bowel syndrome, and diverticular disease. These findings identify Mg homeostasis as a key regulator of gut health and highlight Mg supplementation as a potential strategy to counteract age‐related gut dysfunction.

## Graphical abstract

Aging is associated with a gut‐specific decline in magnesium (Mg), which accelerates intestinal aging and aggravates colitis in aged mice. Mechanistically, Mg deficiency reshapes the phosphoproteome and *N*‐glycoproteome, destabilizing adhesion complexes. Higher dietary Mg intake (334.7–420.0 mg/day) was associated with lower gut disorder risk in the UK Biobank.

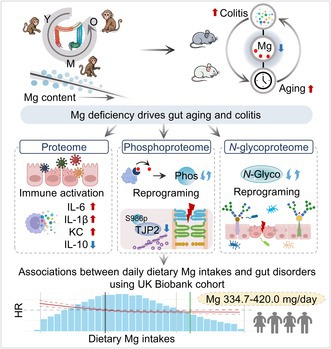

## 1 Introduction

Aging orchestrates multiorgan functional decline (Ding et al. 2025), with the dysfunction of gut showing noteworthy impacts on systemic health (DeJong et al. 2020). As the body's largest endocrine and immune interface, the intestinal epithelium maintains a delicate balance between nutrient absorption and barrier defense (Gribble and Reimann 2019). This equilibrium deteriorates with age, manifesting as increased epithelial permeability, microbial dysbiosis, and low‐grade chronic inflammation (Xiao et al. 2025), processes that not only drive local pathologies (e.g., inflammatory bowel diseases [IBD], colorectal cancer) but also fuel neurodegeneration and metabolic disorders through gut‐brain and gut‐systemic axes (DeJong et al. 2020; Kadyan et al. 2025). Although these findings underscore the pivotal impacts of gut homeostasis on health, the drivers of gut functional decline and related disorders remain incompletely characterized.

Emerging evidence establishes metal ions as a fundamental layer of metabolic regulation in aging (Aron et al. 2025; Morel et al. 2022; Xia et al. 2024). Metal ions serve as cofactors mediating diverse physiological processes in humans, like nerve function (Chen et al. 2025), enzyme activities (Rodriguez et al. 2025), antimicrobial activity (Frei et al. 2023), hormonal balance (Stevenson et al. 2019), immune response (Mu et al. 2021), and various intra‐ and inter‐cellular signaling pathways (Rodriguez et al. 2025). Certain well‐known cell death pathways, such as ferroptosis (Fang et al. 2023) and cuproptosis (Tsvetkov et al. 2022), involve the active participation of metal ions such as iron (Fe) and copper (Cu). In gut physiology, metal elements such as magnesium (Mg) and zinc (Zn) are essential for epithelial cell turnover, immune modulation, and barrier integrity (Lötscher et al. 2022; Shao et al. 2017; Touyz et al. 2024). However, the impacts of age‐related metal element fluctuations on gut physiology and pathologies are not well defined.

Many post‐translational modifications (PTMs), such as phosphorylation and glycosylation, directly or indirectly regulated by metal ions, serve as molecular switches that control essential processes associated with gut homeostasis (Choo et al. 2021; Kudelka et al. 2020). For example, protein phosphorylation regulates processes like epithelial repair (Metwally et al. 2025), immune activation (Liu et al. 2015), and stress responses (Costa‐Mattioli and Walter 2020), whereas glycosylation ensures protein stability, proper folding, and host–microbiota interactions (He et al. 2024). Mg^2+^ serves as a key cofactor for kinases (Kanellopoulou et al. 2019) and glycosyltransferases (Durin et al. 2023), whereas Zn^2+^ supports the activity of histone deacetylases (Porter and Christianson 2019). Dysregulation of these metal ions may consequently influence PTM landscapes and their associated pathways. Despite this mechanistic plausibility, how age‐associated metal ion dyshomeostasis reshapes global PTM networks to drive gut aging and related diseases remains an important but unanswered question in the field.

**FIGURE 1:**
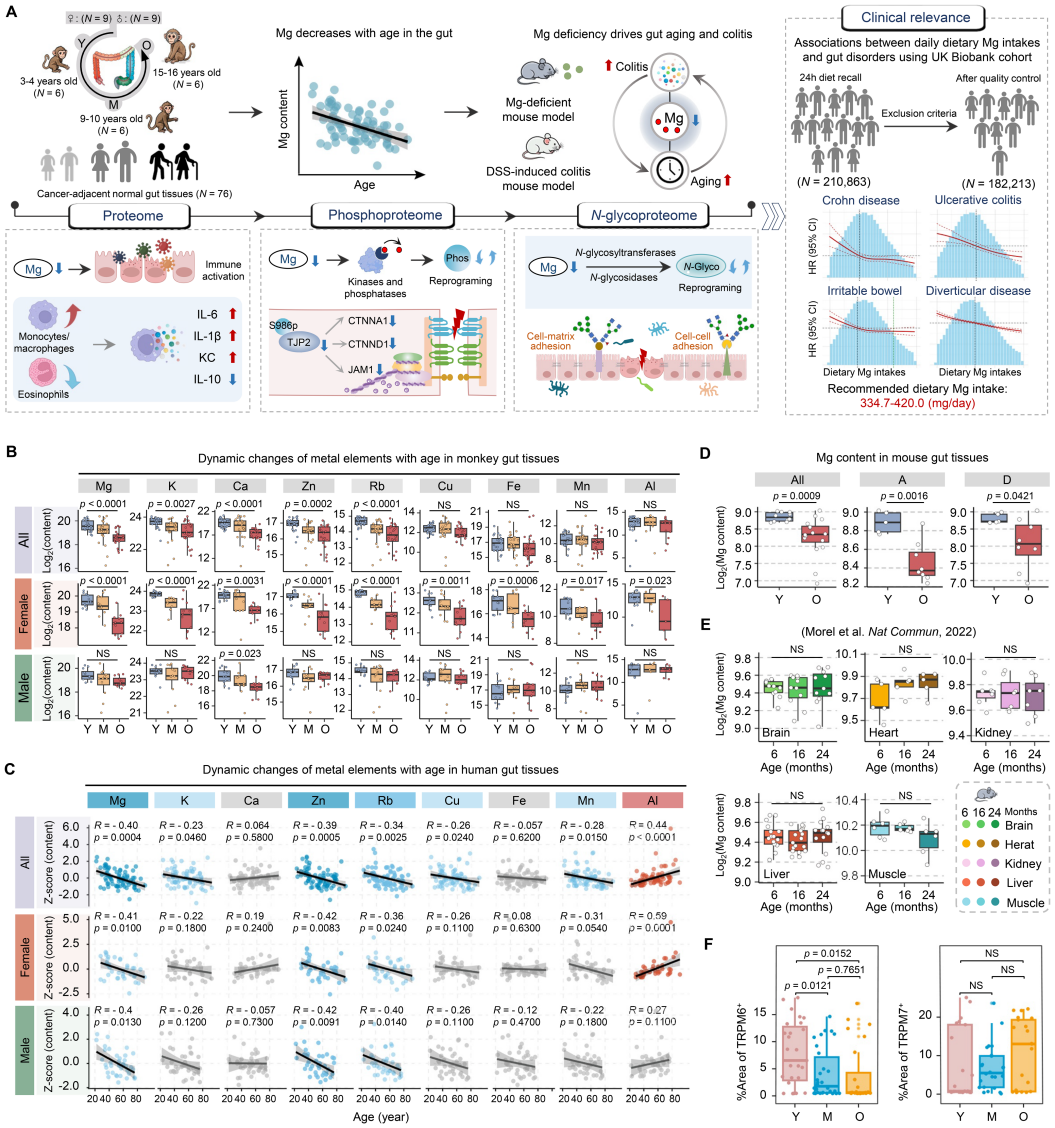
Magnesium decreases with age in the gut tissues across species. (A) Overview of the study design. (B) Box plot displaying the levels of metal elements across three age groups in monkey gut tissues (one‐way ANOVA, median ± quartiles). (C) Scatter plot showing the Spearman's correlations between metal elements levels and age in human gut tissues. (D) Box plot displaying the levels of magnesium (Mg) across two age groups in mouse gut tissues (two‐sided Student's *t*‐test, median ± quartiles). (E) Box plot displaying the levels of Mg across three age groups in mouse five (brain, heart, kidney, liver, and muscle) tissues (two‐sided Student's *t*‐test, median ± quartiles). (F) Quantification of TRPM6 and TRPM7 expression in monkey gut tissues (two‐sided Student's *t*‐test, median ± quartiles). Raw and processed data for drawing are provided in Appendix S1: Source Data Figure 1. A, ascending colon; All, all samples; D, descending colon; M, middle‐aged; NS, non‐significant; O, old; Y, young.

To address these gaps, we systematically explored metal element dynamics and identified Mg^2+^ as a master regulator of gut aging and inflammation. Using multi‐omics analyses and animal models revealed that age‐related Mg deficiency extensively drives the phosphoproteome and *N*‐glycoproteome remodeling, contributing to epithelial barrier dysfunction and gut inflammation. Strikingly, large‐scale analysis of UK Biobank data (*n* = 182,213) discovered that maintaining optimal dietary Mg intake (334.7–420.0 mg/day) significantly reduces the incidence of IBDs and other gut disorders (Figure 1A). These findings position Mg homeostasis as a central regulator of gut health and provide a molecular basis for nutritional interventions to prevent age‐associated intestinal decline and diseases.

## 2 Results

### 2.1 Intestinal Magnesium Decreases With Age Across Species

To explore age‐related changes in the levels of intestinal metal elements, we analyzed gut tissues from multiple species. In non‐human primates, we collected 72 fresh large intestine samples from 18 crab‐eating monkeys divided into three age groups (young: 3–4 years; middle‐aged: 9–10 years; and old: 15–16 years), with balanced sex representation (Figure 1A). For human samples, we obtained 76 distal normal gut tissues from treatment‐naive CRC patients (age range: 22–91 years), equally distributed between proximal and distal regions and matched by sex.

Using inductively coupled plasma mass spectrometry (ICP‐MS), we quantified nine metal elements including Mg, Zn, Cu, Fe, potassium (K), calcium (Ca), rubidium (Rb), manganese (Mn), and aluminum (Al). In monkeys, Mg, K, Ca, Zn, and Rb levels showed significant age‐dependent declines (ANOVA, *p* < 0.05), with females exhibiting more obvious reductions (Figure 1B). Human tissues displayed similar patterns, with Mg, K, Zn, Rb, Cu, and Mn concentrations negatively correlating with age. Notably, Mg, Zn, and Rb significantly decreased in both sexes (Figure 1C). Among the metal elements, Mg exhibited the most consistent age‐associated decline in monkeys and humans. Mouse studies also confirmed this pattern, with significantly lower Mg levels in aged colon, whereas no comparable decrease was observed in the small intestine (Figure 1D and Figure S1A). Importantly, analysis of published mouse metallomic data revealed stable Mg concentrations in other major organs (brain, heart, liver, kidney, muscle) during aging (Morel et al. 2022) (Figure 1E). Additionally, as one of the major storage of Mg^2+^ in the body, we also observed no significant difference in total bone Mg^2+^ content between young and aged human samples (Figure S1B), demonstrating that intestinal Mg declines with age are a tissue‐specific phenomenon rather than systemic deficiency.

To gain insights into the underlying mechanisms associated with age‐related decline in Mg levels, we examined the expression of Mg^2+^ transporters based on our proteomic dataset, such as MAGT1, CNNM4, SLC41A3, and MMGT1, and found that SLC41A3 downregulated with age showed strong positive correlation with intestinal Mg levels (Figure S1C). This is consistent with a previous finding that *Slc41a3* ^−/−^ mice develop hypomagnesemia (de Baaij et al. 2016). Moreover, MAGT1 and CNNM4 also decreased with age, although without statistical significance. Given that intestinal Mg absorption is predominantly mediated by the epithelial Mg^2+^ channels TRPM6 and TRPM7 (Trapani et al. 2018), we further assessed their expression using immunohistochemical analysis. Notably, TRPM6 expression in the intestinal epithelium was significantly reduced with aging, whereas TRPM7 expression did not exhibit a significant age‐associated change (Figure 1F and Figure S1D,E). These results suggest that impaired expression of Mg transporters may contribute to the decline of intestinal Mg levels during aging.

### 2.2 Dietary Magnesium Depletion Drives Gut Aging in Aged Mice

**FIGURE 2:**
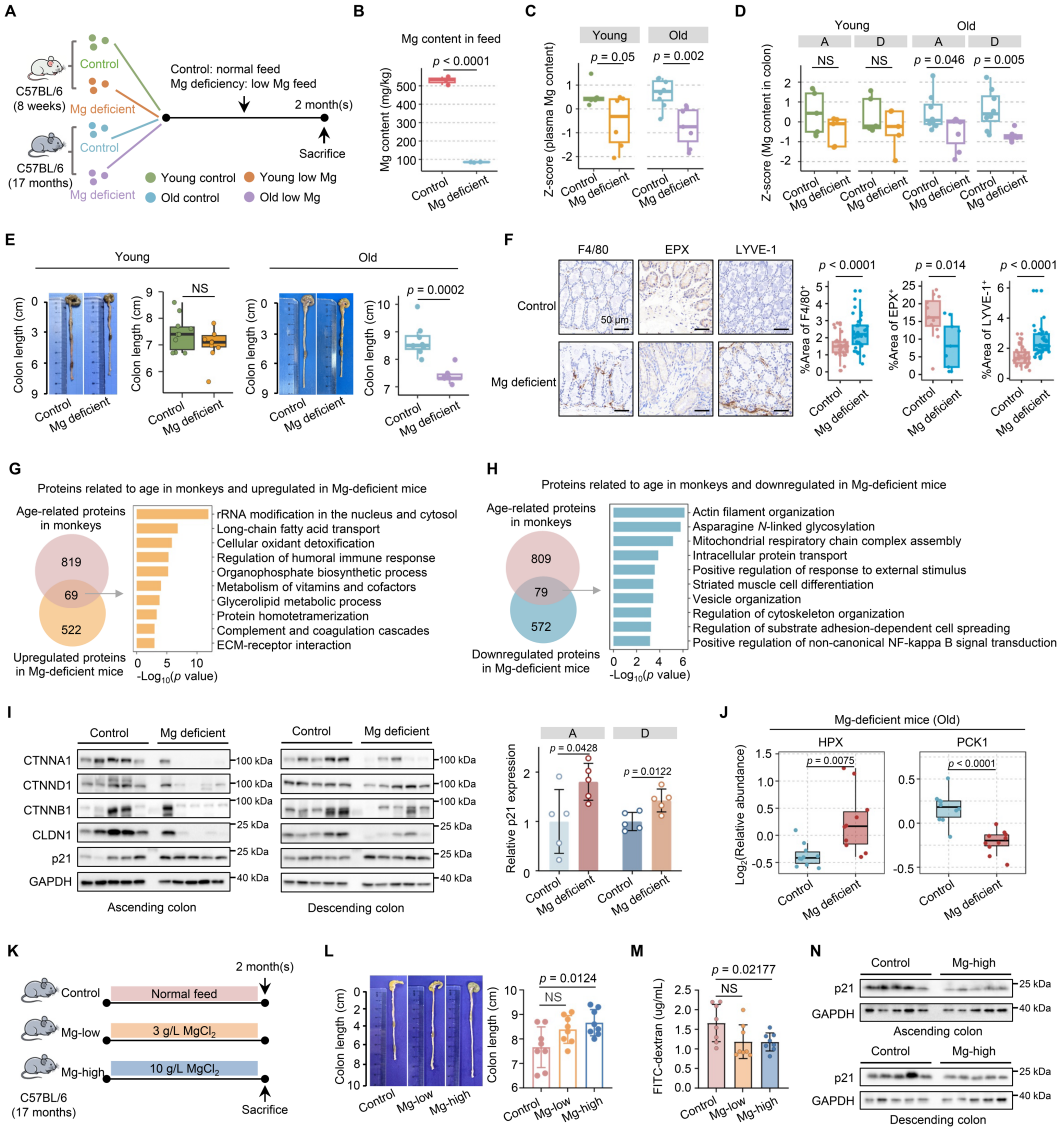
Dietary magnesium depletion drives gut aging in aged mice. (A) Schematic of the experimental regimen. (B) Box plot displaying Mg content in control and Mg‐deficient diets (two‐sided Student's *t*‐test, median ± quartiles). (C) Box plot of scaled plasma Mg levels in young and old mice under normal and Mg‐deficient diets (two‐sided Student's *t*‐test, median ± quartiles). (D) Box plots of scaled Mg levels in the ascending and descending colon of young and old mice under normal and Mg‐deficient diets (two‐sided Student's *t*‐test, median ± quartiles). (E) Representative pictures of mouse colon (left), and statistical analysis of colon length (right) (two‐sided Student's *t*‐test, median ± quartiles). (F) Representative immunohistochemistry images (left) and quantification (right) of F4/80, EPX and LYVE‐1 expression in colons from control and Mg‐deficient aged mice. Scale bars, 50 μm (two‐sided Student's *t*‐test, median ± quartiles). (G, H) Venn diagram displaying overlap between age‐related proteins in monkeys (one‐way ANOVA, *p* < 0.05) and proteins upregulated (G) or downregulated (H) in Mg‐deficient mice (two‐sided Student's *t*‐test, *p* < 0.05). Enriched pathways are shown on the right. (I) Immunoblotting (left) and quantification (right) of p21 and some cell–cell adhesion proteins expression in ascending and descending colon from control and Mg‐deficient old mice (two‐sided Student's *t*‐test, mean ± SD). (J) Box plot displaying HPX and PCK1 levels in control and Mg‐deficient mice (two‐sided Student's *t*‐test, median ± quartiles). (K) Schematic of the experimental regimen. (L) Representative pictures of mouse colon (left), and statistical analysis of colon length (right) (two‐sided Student's *t*‐test, mean ± SD). (M) Box plot displaying the serum FITC‐dextran concentration of control and Mg‐treated old mice (two‐sided Student's *t*‐test, mean ± SD). (N) Immunoblotting of p21 expression in ascending and descending colon from control and Mg‐treated old mice. A, ascending colon; D, descending colon; NS, non‐significant. Raw and processed data and unprocessed images for drawing are provided in Appendix S1: Source Data Figure 2 and Source Image Figure 2.

Proteomic analysis of monkey revealed proteins significantly associated with Mg levels and age were closely related to immune in both females and males (Figure S2A,B). Notably, the number of proteins correlated with Mg content and aging was substantially higher in females than in males, suggesting that Mg‐related regulatory effects may be more pronounced in females. Based on this observation, to further assess the physiological impact of age‐related Mg decline on intestinal aging, we generated a dietary Mg‐deficiency model in young (8 weeks) and aged (17 months) female mice (Figure 2A,B and Figure S2C). ICP‐MS analysis confirmed significant reductions of Mg content in plasma and colonic tissues of aged Mg‐deficient mice (Figure 2C,D). Colon shortening, a hallmark of intestinal aging, occurred exclusively in aged Mg‐deficient mice (two‐sided Student's *t*‐test, *p* = 0.0002), whereas the young mice remained less affected (Figure 2E), suggesting the aged gut is more susceptible to micronutrient imbalances. Immune profiling using mMCP‐counter revealed that Mg depletion triggered pro‐inflammatory remodeling in aged mice, characterized by increased infiltration of macrophages and lymphocytes alongside reduced eosinophils (Figure S2D). These findings were further validated by immunohistochemical analysis, which confirmed consistent changes in the abundance and distribution of these immune cell populations in colonic tissues (Figure 2F). These changes mirror immunosenescence patterns in human gut aging (Choi and Augenlicht 2024), positioning Mg deficiency as a potential accelerator of age‐related immune dysfunction.

Our proteomic analysis revealed molecular reprogramming in aged Mg‐deficient mice, identifying 591 upregulated and 651 downregulated proteins (*p* < 0.05, ratio [Mg deficient/control] > 1.2 or < 0.83) (Figure S2E). By overlapping these changes with age‐related proteins from monkeys, we uncovered conserved pathways linking Mg deficiency to gut aging (Figure 2G,H). Importantly, sex‐stratified analysis revealed that these conserved associations were also observed in females (Figure S2F,G). The upregulated proteins were mainly enriched in three key processes, including immune responses, metabolic reprogramming, and extracellular matrix (ECM) remodeling, all hallmarks of aged tissues (Kroemer et al. 2025). In contrast, the downregulated proteins were enriched in the structural and functional maintenance pathways including actin cytoskeleton organization, protein glycosylation, and mitochondrial respiration (Figure 2G,H). These coordinated changes are crucial for accelerated gut aging. For example, impaired mitochondrial function reduces cellular energy production (Zong et al. 2024), whereas disrupted glycosylation may compromise protein quality control (He et al. 2024). Alongside, cytoskeletal disorganization weakens epithelial integrity (Kang et al. 2023), and ECM remodeling promotes tissue stiffness (Stearns‐Reider et al. 2017). Immunoblot analysis confirmed a reduction in cell–cell adhesion proteins in aged Mg‐deficient mice. In parallel, the senescence marker p21 was markedly elevated in response to Mg deficiency (Figure 2I). Furthermore, the well‐known pro‐aging gene *HPX* was upregulated and the anti‐aging gene *PCK1* was downregulated in Mg‐deficient mice, respectively (Wang et al. 2024) (Figure 2J). These Mg deficiency‐induced changes precisely target pathways known to decline with normal aging, suggesting Mg homeostasis serves as a key regulator of gut health. To further explore the functional relevance of Mg status in the aged intestine, we performed Mg supplementation in aged mice (Figure 2K). Mg supplementation was associated with increased colon length and markedly improved intestinal barrier integrity, as evidenced by reduced intestinal permeability (Figure 2L,M). In parallel, the expression of the senescence marker p21 was significantly decreased (Figure 2N). Together, these findings suggest that maintenance of Mg homeostasis may contribute to the preservation of intestinal structure and function during aging.

Given the essential role of Mg^2+^ as a cofactor for phosphorylation and glycosylation enzymes, we investigated how Mg deficiency contributes to gut aging through modulating these PTMs. The reliability of our phosphoproteomic and *N*‐glycoproteomic data were confirmed through rigorous quality control (QC) (Figure S3A,B). Sex‐stratified analyses further showed results consistent with the proteomic data in monkeys; phosphoproteomic and *N*‐glycoproteomic analyses revealed that the numbers of phosphosites and *N*‐glycopeptides significantly associated with Mg content and age were markedly higher in females than in males (Figure S3C,D). Pathway enrichment analysis in females indicated that Mg‐ and age‐associated phosphosites were predominantly involved in cell adhesion‐related pathways, whereas *N*‐glycopeptides were mainly enriched in ECM and immune‐associated pathways. Notably, these pathways are closely implicated in the regulation of aging processes, suggesting that Mg may influence aging by modulating the levels of PTMs.

**FIGURE 3:**
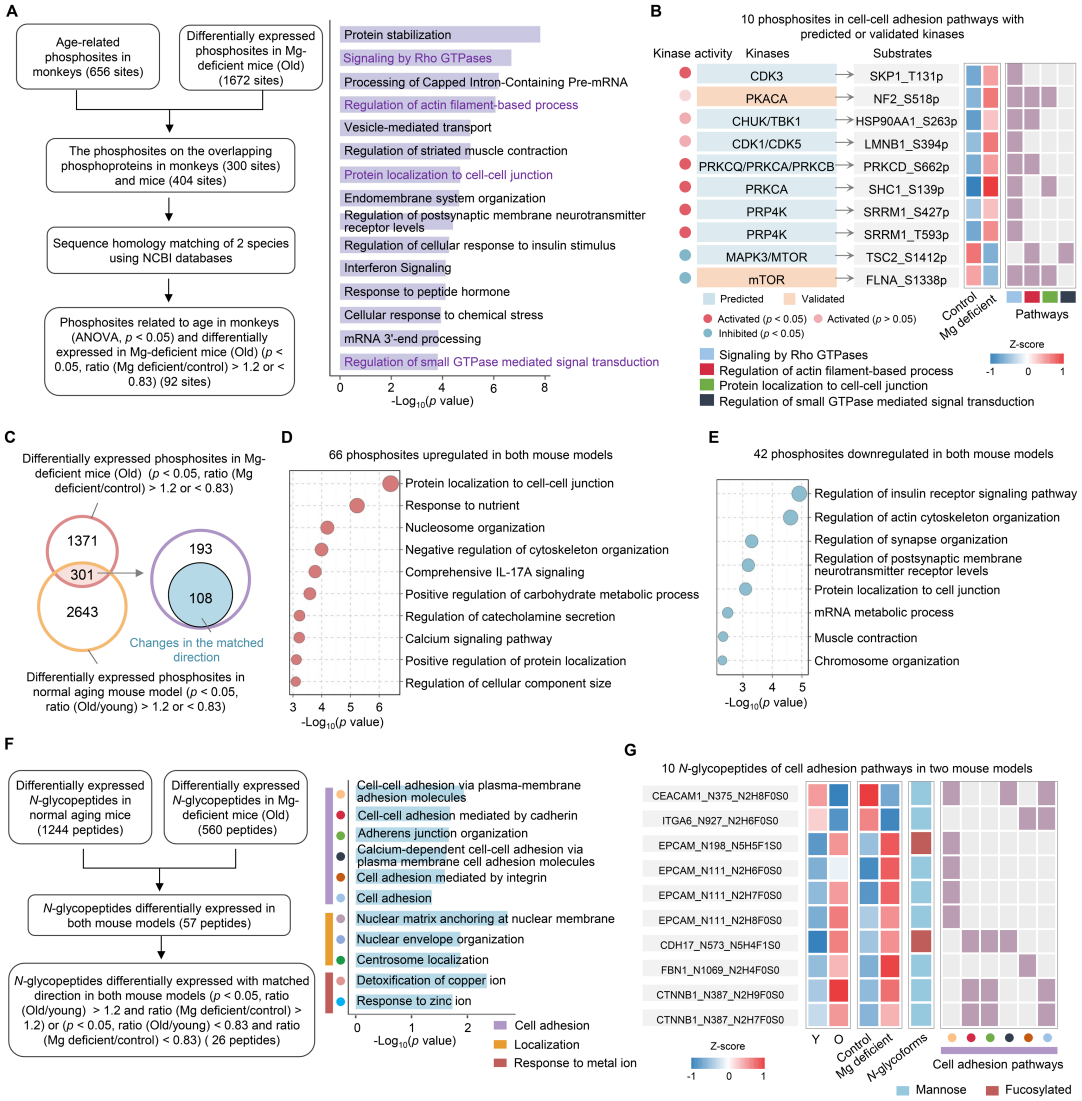
Dietary magnesium depletion causes PTM reprogramming in aging gut. (A) Flow chart of screening phosphosites with age‐related changes in monkeys and differential expression in Mg‐deficient mice (left). Enriched pathways based on these phosphoproteins are shown in the right panel. (B) Heatmap of 10 phosphosites in cell–cell adhesion pathways with predicted or validated kinases in control and Mg‐deficient mice (middle). Predicted or validated kinases and their corresponding kinase activities are shown on the left, with the associated cell–cell adhesion‐related pathways on the right. (C) Venn diagram displaying overlapping phosphosite changes with matched changing patterns in Mg‐deficient and natural aging mouse models (two‐sided Student's *t*‐test, *p* < 0.05). (D, E) The pathways enriched using the phosphoproteins of 66 phosphosites upregulated (D) and 42 phosphosites downregulated (E) in both Mg‐deficient and natural aging mice. (F) Flow chart of screening *N*‐glycopeptides changes in matched changing patterns in Mg‐deficient and natural aging mice (two‐sided Student's *t*‐test, *p* < 0.05) (left), with enriched pathways based on the screened *N*‐glycoproteins shown on the right. (G) Heatmap of 10 *N*‐glycopeptides in cell–cell adhesion pathways in both Mg‐deficient and natural aging mice (left). The corresponding *N*‐glycan composition is shown in the middle panel. The involved cell–cell adhesion‐related pathways are shown on the right. Raw and processed data for drawing are provided in Appendix S1: Source Data Figure 3. O, old; Y, young.

In addition, further analysis showed phosphoproteomics identified 1672 phosphosites significantly changed in Mg‐deficient mice (*p* < 0.05, ratio [Mg deficient/control] > 1.2 or < 0.83) (Figure S4A). Kinase‐substrate enrichment analysis (KSEA) revealed a complex pattern of kinase activity changes, with both increased (e.g., SRPK1, PRP4K and CDK3) and decreased (e.g., MAPK10, mTOR and ERK2) kinase activities observed (Figure S4B), demonstrating the central role of Mg in phosphorylation network regulation. The observed bidirectional kinase activity regulation may stem from distinct mechanisms. For example, reduced Mg availability may directly impair kinase/phosphatase activity via reduced Mg·ATP availability, or indirectly activate the compensatory Ca^2+^‐dependent signaling (Gusarova et al. 2011; Oost et al. 2023). Among the significantly altered phosphosites, 92 were both age‐related in monkeys and Mg‐responsive in mice (Figure 3A). The proteins harboring these sites were primarily enriched in pathways such as Rho GTPase signaling, actin filament regulation, protein localization to cell–cell junctions, and small GTPase–mediated signal transduction, all of which are essential for maintaining epithelial integrity (Figure 3A). Further analysis of these pathways identified 35 phosphosites including 10 with predicted kinases. For instance, PKACA was validated to phosphorylate NF2 at Ser518, a modification known to regulate cell–cell adhesion (Petrilli and Fernández‐Valle 2016), suggesting that Mg may affect gut aging by modulating kinase activities and associated phosphorylation of adhesion proteins (Figure 3B). Consistently, we identified 108 phosphosites showing consistent changes in both natural aging and Mg‐deficient mouse models, reinforcing that Mg impacts the phosphorylation of cell–cell adhesion pathways (Figure 3C–E). These findings indicate that Mg deficiency may disrupt intestinal barrier function by altering the phosphorylation of adhesion proteins.

Moreover, we identified 560 differentially expressed *N*‐glycopeptides in Mg‐deficient mouse models (*p* < 0.05; ratio [Mg deficient/control] > 1.2 or < 0.83) (Figure S4C). Specifically, Mg‐deficient mice showed increased fucosylation and hybrid/complex glycans in upregulated peptides, whereas downregulated peptides were enriched in sialylated and mannose‐type glycans (Figure S4D). These changes likely impair protein function by affecting folding, stability, and cell–surface interactions (Duran‐Romaña et al. 2024). Cross‐species analysis with aging monkeys showed that both Mg deficiency and natural aging similarly affect *N*‐glycosylation in cell adhesion pathways (Figure S4E,F). Consistently, the proteins with *N*‐glycopeptides showing consistent changes in both natural aging mice and Mg‐deficient mice were also enriched in cell adhesion pathways (Figure 3F), supporting the potential link between Mg‐dependent *N*‐glycosylation and intestinal barrier integrity. Moreover, most adhesion‐related *N*‐glycopeptides were mannose‐type, with a smaller fraction being fucosylated, and exhibited bidirectional changes in response to Mg deficiency (Figure 3G). For example, the level of mannose‐type of ITGA6_N927_N2H6F0S0 decreased, whereas the fucosylated‐type of CDH17_N573_N5H4F1S0 increased, changes that may weaken epithelial adhesion. Mechanistically, we assumed that Mg deficiency impairs both *N*‐glycosyltransferases and *N*‐glycosidases (Figure S4G), disrupting glycan biosynthesis and turnover, which may weaken epithelial barrier function and accelerate gut aging. These results indicate that Mg influences gut aging, at least in part, through modulating protein phosphorylation and *N*‐glycosylation involved in cell adhesion pathways. Dysregulated PTMs may disrupt epithelial architecture and barrier integrity, facilitating age‐associated decline in gut function.

### 2.3 Dietary Magnesium Deficiency Exacerbates Gut Inflammation

**FIGURE 4:**
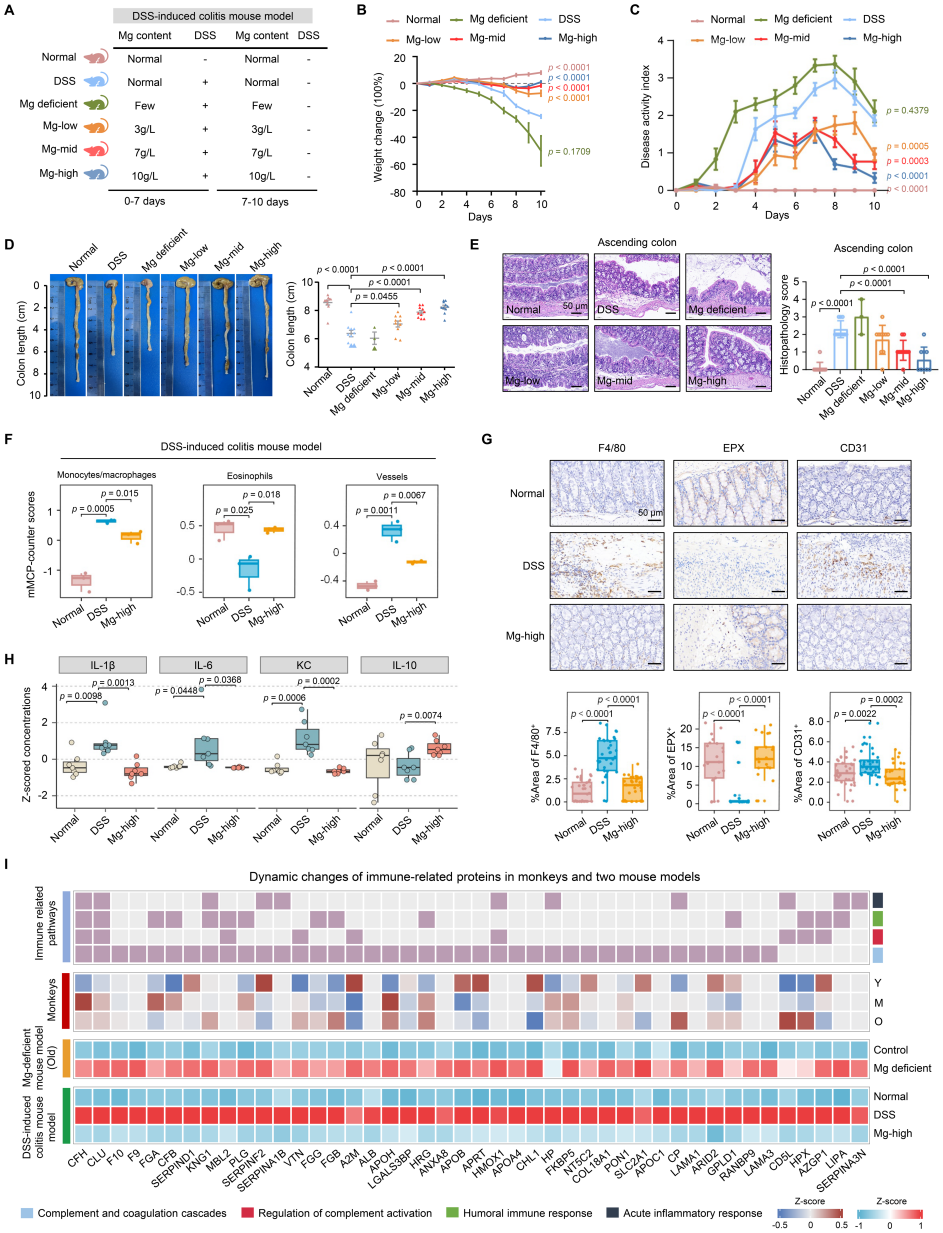
Dietary magnesium deficiency exacerbates gut inflammation. (A) Schematic of the experimental regimen. (B) Daily mouse body weight (two‐sided Student's *t*‐test, mean ± SEM). (C) Disease activity index (DAI) determined by weight loss, stool consistency and bleeding (two‐sided Student's *t*‐test, mean ± SEM). (D) Statistical analysis of colon length (two‐sided Student's *t*‐test, mean ± SEM). (E) Representative H&E images of ascending colons. Scale bars, 50 μm. Box plot illustrates statistical results of histopathology score (0 = none; 1 = very mild; 2 = mild; 3 = moderate; 4 = severe) (two‐sided Student's *t*‐test, mean ± SD). (F) Box plots of mMCP‐counter scores in DSS‐induced colitis mouse model among the normal, DSS, and Mg‐high groups (two‐sided Student's *t*‐test, median ± quartiles). (G) Representative immunohistochemistry images (up) and quantification (down) of F4/80, EPX and LYVE‐1 expression in DSS‐induced colitis mouse model among the normal, DSS, and Mg‐high groups. Scale bars, 50 μm (two‐sided Student's *t*‐test, median ± quartiles). (H) Box plots of inflammatory cytokine levels among the normal, DSS, and Mg‐high groups (two‐sided Student's *t*‐test, median ± quartiles). (I) Heat map showing the dynamic changes of immune‐related proteins in monkeys, Mg‐deficient mice, and DSS‐induced colitis mice. The involved immune‐related pathways are shown in the upper panel. M, middle‐aged; O, old; Y, young. Raw and processed data and unprocessed images for drawing are provided in Appendix S1: Source Data Figure 4 and Source Image Figure 4.

Gut aging is frequently accompanied by chronic inflammation. To link Mg deficiency, gut aging, and colitis, we used DSS‐induced colitis mice with a normal chow diet, Mg‐deficient chow diet, and Mg‐supplemented chow diet (low/mid/high), and found that Mg supplementation reduced inflammation and restored colon structure (Figure 4A–D and Figure S5A,B). Histology showed Mg deficiency worsened epithelial damage and immune infiltration, whereas Mg supplementation alleviated these effects (Figure 4E and Figure S5C). mMCP‐counter analysis and immunohistochemical staining revealed that Mg repletion decreased macrophages but increased eosinophils (Figure 4F,G), and shifted cytokines toward an anti‐inflammatory profile (Figure 4H), indicating Mg restriction disrupts immune homeostasis while supplementation restores balance.

To further elucidate the role of Mg in the process of colitis, we performed proteomic profiling. Our integrated analysis of Mg‐deficient and DSS‐induced colitis models identified 65 proteins showing consistent upregulation in inflammatory conditions that were compromised by Mg supplementation. Moreover, these proteins were predominantly involved in immune activation pathways, including complement activation and acute inflammatory responses (Figure S5D), consistent with the cytokine and immune cell alterations (Figure 4F–H). Notably, 44 of these proteins were directly implicated in immune regulation, with cross‐species validation showing 20, including FGB, CP, and CD5L, exhibiting parallel age‐related decline with decreasing Mg levels in monkeys (Figure 4I), suggesting a conserved mechanism linking Mg deficiency to immune dysregulation during aging. Conversely, 59 proteins were decreased under Mg deficiency and in colitis mouse models but increased progressively with Mg supplementation. These proteins were primarily associated with proteostasis, particularly protein trafficking and glycosylation processes (Figure S5D). The consistent enrichment of phosphorylation‐ and glycosylation‐related pathways across both protein groups underscores the role of Mg in regulating PTMs that preserve gut homeostasis.

**FIGURE 5:**
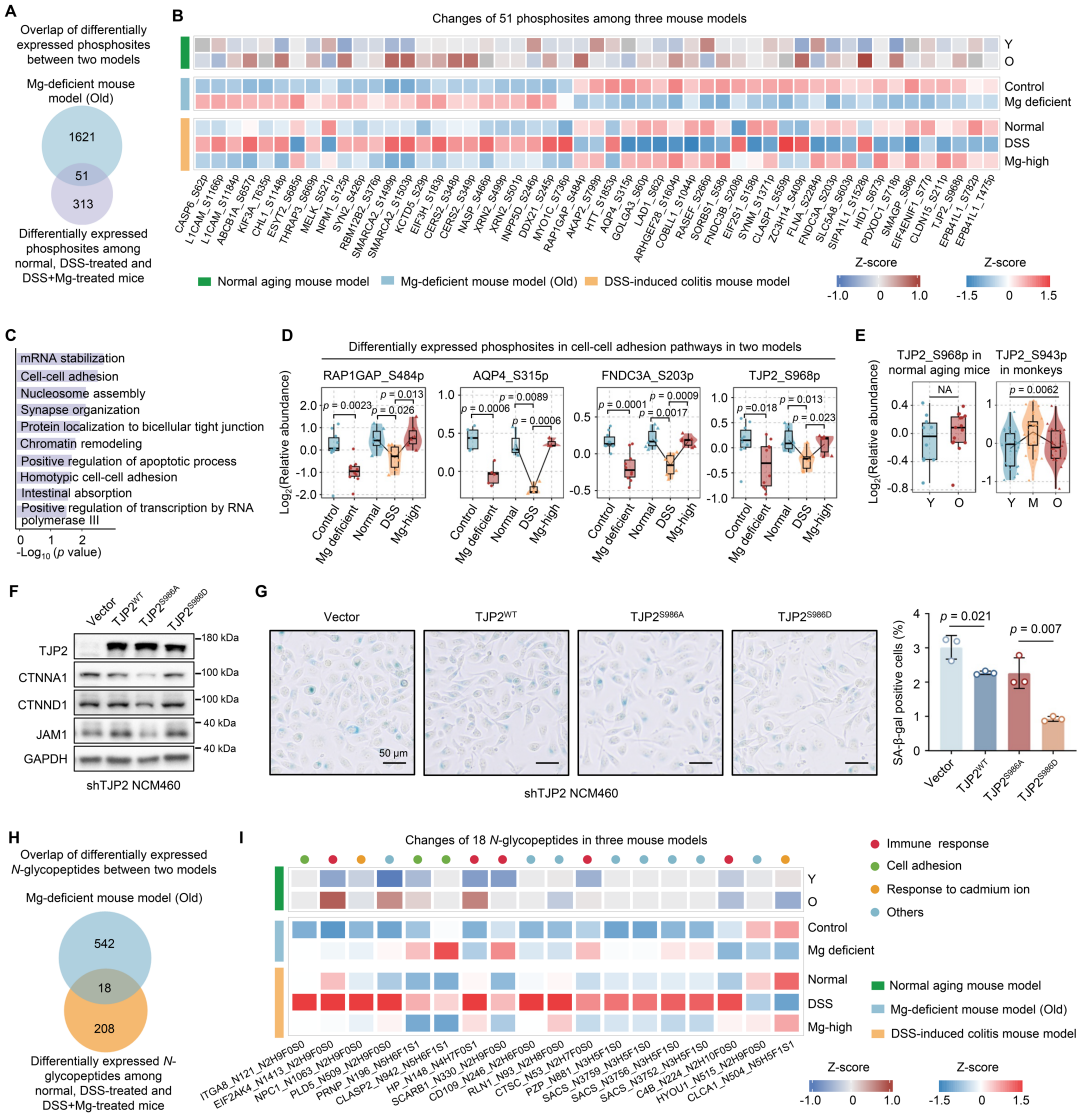
Magnesium deficiency causes PTM reprogramming in colitis. (A) Venn diagram displaying overlapping phosphosites between Mg‐deficient and DSS‐induced colitis mouse models (two‐sided Student's *t*‐test, *p* < 0.05). (B) Heatmap showing the changes of 51 overlapping phosphosites in natural aging, Mg‐deficient and DSS‐induced colitis mice. (C) The pathways enriched using the corresponding phosphoproteins of 51 overlapping phosphosites. (D) Box plots of differentially expressed phosphosites in cell–cell adhesion pathways in Mg‐deficient and DSS‐induced colitis mice (two‐sided Student's *t*‐test). (E) Box plots displaying the levels of TJP2^S968p^ in natural aging mice (two‐sided Student's *t*‐test) and monkeys (one‐way ANOVA). (F) Immunoblotting showing the expression of some cell–cell adhesion proteins in human NCM460 cell line stably transfected with TJP2^WT^, TJP2^S986A^, or TJP2^S986D^ plasmid. (G) Representative images of senescence‐associated β‐galactosidase (SA‐β‐gal) staining in cells transfected with TJP2^WT^, TJP2^S986A^, or TJP2^S986D^ plasmid (left). Box plot illustrating statistical results of SA‐β‐gal positive cells (right) (two‐sided Student's *t*‐test, mean ± SD). Scale bars, 50 μm. (H) Venn diagram of the overlapping *N*‐glycopeptides between Mg‐deficient and DSS‐induced colitis mouse models (two‐sided Student's *t*‐test, *p* < 0.05). (I) Heatmap displaying the changes of 18 overlapping *N*‐glycopeptides in natural aging, Mg‐deficient and DSS‐induced colitis mouse models. M, middle‐aged; O, old; Y, young. Raw and processed data and unprocessed images for drawing are provided in Appendix S1: Source Data Figure 5 and Source Image Figure 5.

To elucidate how Mg alleviates colitis through PTMs, we integrated phosphoproteomic and *N*‐glycoproteomic data from DSS‐induced colitis and Mg‐deficient mouse models. Our analysis identified numerous phosphosites that were either decreased in colitis but restored by Mg supplementation, or increased in colitis but suppressed under Mg treatment (Figure S6A,B). Consistent with the above findings (Figure 3A), these phosphorylation changes also primarily involved cell adhesion pathways including GTPase regulation and cytoskeletal organization (Figure S6A,B). Overlapping these differential phosphosites with those identified in Mg‐deficient mice revealed 51 sites significantly altered in both models (Figure 5A). Many of these phosphosites were located on proteins previously implicated in intestinal aging and colitis, such as SLC5A8 (Gurav et al. 2015), TJP2 (Odenwald and Turner 2017), CERS2 (Oertel et al. 2017), suggesting Mg modulates gut function through phosphorylation of structural proteins (Figure 5B). Moreover, 41 of the 51 phosphosites were detected in the natural aging mouse model, with 20 phosphosites displaying consistent changes across Mg‐deficient, DSS‐induced colitis, and natural aging models, indicating these Mg‐regulated phosphorylation may contribute to both gut aging and colitis (Figure 5B). Although some phosphosites showed consistent directional changes in the Mg‐deficient and DSS‐induced colitis models but displayed opposite trends in the natural aging model such as RAP1GAP^S484p^. These differences suggest that phosphosites regulation in aging does not only reflect Mg availability but may also arise from broader age‐dependent physiological remodeling. Aging is known to introduce additional regulatory influences including chronic inflammation, mitochondrial dysfunction, and altered kinase–phosphatase activity (Barbagallo et al. 2021; Dominguez et al. 2024; Killilea and Maier 2008). In contrast, increasing evidence indicates that Mg can directly regulate phosphorylation signaling, contributing to Mg‐responsive phosphoproteome remodeling (Zhang et al. 2024). Therefore, the patterns observed across models likely reflect two interacting mechanisms: phosphosites that respond primarily to Mg fluctuations and others influenced by aging‐related signaling rewiring.

Pathway analysis of proteins containing these 51 phosphosites also revealed their primary involvement in cell–cell adhesion mechanisms (Figure 5C). Notably, TJP2^S968p^ (mouse) was significantly downregulated under Mg deficiency and restored with Mg supplementation (Figure 5D), but it didn't show consistent age‐related changes (Figure 5E). The conserved sites are TJP2^S943^ in monkey and TJP2^S986^ in human. Functional studies demonstrated that phospho‐mimetic TJP2^S986D^ in human NCM460 cell line enhanced adhesion protein expression (CTNNA1, CTNND1, JAM1) (Figure 5F) and reduced cellular senescence (Figure 5G), suggesting its protective role during gut aging. TJP2^S968p^ is regulated by Mg yet also affected by other unknown factors, indicating the complex regulatory mechanism of phosphorylation.

Further analysis of *N*‐glycoproteomic data identified two distinct groups of *N*‐glycopeptides showing reciprocal changes between DSS‐induced colitis and Mg supplementation (Figure S6C,D). Pathway analysis revealed these glycoproteins were also predominantly involved in cell adhesion processes including integrin‐mediated adhesion and cell–matrix interactions (Figure S6C,D). Glycan composition analysis showed that Module1 was characterized by high mannose/core fucosylation, with a higher proportion of fucosylated and fucosylated‐sialylated structures, whereas Module2 was enriched in sialylated and hybrid/complex *N*‐glycans (Figure S6E). These differences persisted when focusing on *N*‐glycopeptides within cell adhesion pathways, suggesting that Mg supplementation remodels the glycan architecture of adhesion proteins (Figure S6E). Moreover, we observed coordinated changes in *N*‐glycosylation of receptor‐ligand pairs involved in cell–cell communication (Figure S6F), indicating Mg may regulate intercellular signaling through *N*‐glycosylation. Notably, Mg deficiency reduced the levels of some *N*‐glycosyltransferases and *N*‐glycosidases, whereas supplementation restored their levels (Figure S6G), indicating that Mg modulates *N*‐glycosylation not only through regulating enzyme activities but also affecting enzyme expression. We identified 18 *N*‐glycopeptides consistently altered in both Mg deficiency and colitis models (Figure 5H), primarily involved in immune response, cell adhesion, and response to cadmium ion (Figure 5I). Importantly, 10 of 11 detectable *N*‐glycopeptides in aging mouse model showed parallel changes, including cell adhesion–related PRNP_N196_N5H6F1S1 and immune‐related HP_N148_N4H7F0S1, confirming the conserved role of these *N*‐glycopeptides during both gut aging and inflammation. Our findings indicate that Mg protects against both inflammatory and age‐related intestinal dysfunction. The convergence on cell adhesion pathways highlights Mg's role in reinforcing epithelial integrity by modulating protein modifications of key structural and signaling components.

### 2.4 Associations Between Dietary Mg Intake and Gut Health

To assess the clinical relevance of our above findings, we analyzed data from the UK Biobank, a large prospective cohort study with comprehensive dietary and health records. Our analysis included 182,213 participants with a 13‐year follow‐up (2009–2022). Baseline characteristics of the participants, according to quintiles of dietary Mg intake, were presented (Table S1).

**FIGURE 6:**
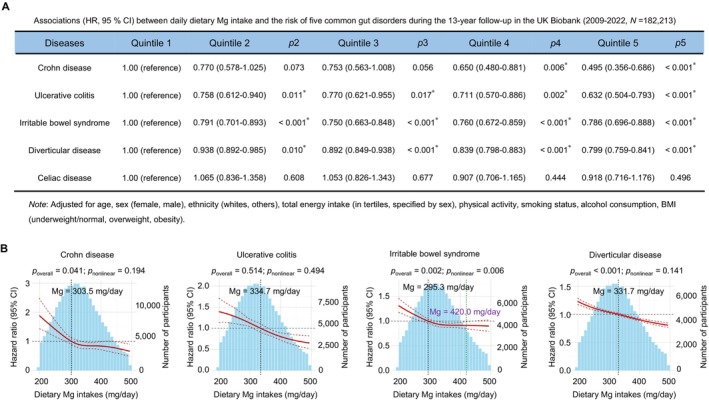
Associations between dietary magnesium intake and gut disorder incidence. (A) Associations (HR, 95% CI) between daily dietary Mg intake and the risk of five common gut disorders in the UK Biobank participants during the 13‐year follow‐up (2009–2022, *N* = 182,213). Multivariable‐adjusted models were adjusted for age, sex (female, male), ethnicity (whites, others), total energy intake (in tertiles, specified by sex), physical activity, smoking status, alcohol consumption, BMI (underweight/normal, overweight, obesity). Significance was calculated using the Cox multivariate proportional hazards regression model. (B) Logistic regression analyses of dietary Mg intake and gut disorder risk, with adjustments for age, sex (female, male), ethnicity (whites, others), total energy intake (in tertiles, specified by sex), physical activity, smoking status, alcohol consumption, BMI (underweight/normal, overweight, obesity). Dietary Mg intake is presented on the *x*‐axis, excluding values outside the 5th and 95th percentiles. Histograms depict the distribution of dietary Mg intake by participants. HRs are represented by solid lines, and the corresponding 95% CIs are depicted by the areas between the dashed lines. CIs, confidence intervals; HRs, hazard ratios. Raw and processed data for drawing are provided in Appendix S1: Source Data Figure 6.

To assess the associations between levels of total Mg and the risk of gut disorders, the Cox multivariate proportional hazards regression model was applied to estimate the hazard ratios (HRs) and 95% confidence interval (CI) after adjusting the various confounding factors including age, sex, ethnicity, total energy intake, smoking status, alcohol consumption and body mass index (BMI). In multivariable‐adjusted Cox analyses comparing the highest versus lowest quintiles of dietary Mg intake, we observed significantly reduced hazards for several gut disorders among individuals with higher Mg consumption. Specifically, the HRs were 0.495 (95% CI: 0.356–0.686, *p* < 0.001) for Crohn's disease, 0.632 (95% CI: 0.504–0.793, *p* < 0.001) for ulcerative colitis, 0.786 (95% CI: 0.696–0.888, *p* < 0.001) for irritable bowel syndrome, and 0.799 (95% CI: 0.759–0.841, *p* < 0.001) for diverticular disease (Figure 6A). Spline dose–response analyses provided additional insights into these associations. Although the overall spline for ulcerative colitis did not reach statistical significance despite noteworthy quintile comparisons, we found significant associations for Crohn's disease (*p*_overall_ = 0.041), irritable bowel syndrome (*p*_overall_ = 0.002; *p*_nonlinear_ = 0.006), and diverticular disease (*p*_overall_ < 0.001) (Figure 6B). The spline models indicated that HRs for all four conditions fell below unity at dietary Mg intakes exceeding 334.7 mg/day and remained protective up to 420.0 mg/day, with confidence intervals for irritable bowel syndrome approaching unity beyond this upper threshold. Based on this, we identified a conservative population‐level intake window of 334.7–420.0 mg/day associated with reduced disease risk across all four intestinal conditions in this cohort. This protective range suggests that maintaining dietary Mg intake within these levels may confer benefits for gut health.

To evaluate the robustness of these associations and address potential clinical heterogeneity, we conducted a series of prespecified sensitivity and subgroup analyses. First, to minimize confounding by poor nutritional status, we excluded 42,595 participants diagnosed with malnutrition at baseline according to the Global Leadership Initiative on Malnutrition (GLIM) criteria. As shown in Figure S7A, the inverse associations between dietary Mg intake and the risks of Crohn's disease, ulcerative colitis, irritable bowel syndrome, and diverticular disease remained statistically significant, indicating that the primary findings were not driven by under‐nutrition.

Furthermore, stratified analyses were performed according to comorbidities status (including hypertension, diabetes, and cardiovascular disease) and the use of common medications (including antihypertensive, diuretic, antidiabetic, antiplatelet, and lipid‐lowering agents). As illustrated in Figure S7B, the associations between higher Mg intake and reduced risk of gut disorders were generally consistent between all subgroups and overall subjects. Hazard ratios and corresponding confidence intervals were comparable between participants with and without comorbidities, as well as between medication users and non‐users, with no evidence of substantial effect modification. Collectively, these analyses support the stability and robustness of the observed associations between dietary Mg intake and gut health across diverse clinical profiles.

## 3 Discussion

This study establishes Mg deficiency as a key driver of gut aging and colitis through multi‐species investigations. Mg deficiency disrupts gut barrier function by impairing proteostasis, promoting inflammation and senescence, and reshaping the phosphoproteome and *N*‐glycoproteome. Analysis of the UK Biobank (*n* = 182,213) confirms that adequate dietary Mg intake (334.7–420.0 mg/day) significantly reduces the risk of Crohn's disease (HR = 0.495), ulcerative colitis (HR = 0.632), and other intestinal disorders. These findings highlight scientific Mg supplementation as a promising strategy to preserve gut barrier integrity, mitigate age‐related functional decline, and prevent intestinal disorders, providing a practical nutritional approach to maintaining gut health across the lifespan.

A notable observation from our data are that young mice fed a Mg‐deficient diet did not show significant reductions in plasma or colonic Mg^2+^ levels, despite a downward trend resembling that of aged mice. This likely reflects the relatively strong regulatory capacity in early life, where adaptive mechanisms, including increased fractional intestinal absorption under low Mg intake and efficient renal reabsorption (Barbagallo et al. 2021; Nie et al. 2024), can partially buffer systemic Mg^2+^ levels despite dietary restriction. With aging, Mg homeostasis is gradually disrupted, as evidenced by reduced intestinal absorption and decreased renal reabsorption capacity (Barbagallo et al. 2021), making older animals more susceptible to measurable tissue‐level Mg^2+^ decline under the same dietary conditions. The absence of structural alterations, such as stable colon length in young mice regardless of diet, further supports the view that young organisms can tolerate and compensate for transient Mg restriction, whereas aging increases vulnerability to Mg^2+^ imbalance.

Metal elements play fundamental roles in regulating PTMs through diverse mechanisms. As essential cofactors, they directly modulate enzyme activities and substrate binding. For example, Mg^2+^ facilitates phosphate transfer in phosphorylation reactions (Nam et al. 2024), Zn^2+^ activates deacetylases controlling epigenetic regulation (Werbeck et al. 2020), and Mn^2+^ is indispensable for glycosyltransferase function in *N*‐glycosylation pathways (De Marco et al. 2025). Our systematic investigation provides animal‐level evidence linking metal element deficiency to PTM dysregulation in gut aging and colitis, establishing a framework for understanding how metal element imbalances contribute to age‐related pathologies through their effects on protein modification networks. Furthermore, overlap analysis revealed that while Mg‐regulated phosphorylation and *N*‐glycosylation have potential effects on numerous distinct pathways, multiple cell adhesion‐related pathways are commonly regulated by both protein modifications, suggesting coordinated functions between phosphorylation and *N*‐glycosylation (Figure S8A).

Our findings show that intestinal Mg levels decline progressively with age, a process that may be driven, at least in part, by altered expression of Mg transporters. Consistent with this, analysis of published datasets revealed a marked reduction in the Mg efflux transporter CNNM4 in patients with ulcerative colitis (Guo et al. 2025; Liu et al. 2025), mirroring our observations in DSS‐induced colitis mice (Figure S8B). CNNM4 mediates basolateral export of Mg^2+^ from intestinal epithelial cells into the circulation, sustaining systemic Mg homeostasis (Yamazaki et al. 2013). Reduced CNNM4 expression observed in both ulcerative colitis patients and aged intestines may represent a compensatory response to local Mg deficiency, in which epithelial cells restrict further export to preserve intracellular Mg. However, this adaptation could impair epithelial repair and barrier integrity, ultimately exacerbating chronic inflammation.

Using the Biobank cohort, we identified an optimal dietary Mg intake range of approximately 334.7–420.0 mg/day that is associated with reduced risks of gut disorders. Although Mg can be obtained through diet, individuals with marked deficiency may require additional supplementation, which necessitates careful consideration of both timing and dosage. Evidence suggests that Mg supplementation initiated in early or mid‐life provides stronger protection for gut homeostasis, whereas late‐life intake is less effective due to reduced absorption efficiency and impaired tissue repair (Adomako and Yu 2024; Barbagallo et al. 2021; Dominguez et al. 2024). Our findings indicate that dietary Mg deficiency exerts a more pronounced impact on gut aging in aged mice, implying that older individuals are particularly sensitive to Mg fluctuations. Given that Mg content declines with age, deficiency being uncommon in youth but increasingly prevalent from mid‐life onward, and that the incidence of gut disorders rises substantially in middle and late life, Mg supplementation during these stages may represent an effective preventive strategy. Likewise, interventions introduced at the onset of inflammation are more likely to confer benefits than those applied at advanced disease stages (Wang et al. 2025). Importantly, dosage must be carefully optimized, as inadequate intake increases inflammatory susceptibility, whereas excessive intake may impose risks on Mg‐sensitive organs such as heart and kidneys (Lötscher et al. 2022; Macías Ruiz et al. 2023). Future clinical studies adopting age‐stratified and dose‐gradient designs will be essential to refine these strategies and guide personalized interventions.

Moreover, our findings disclose that multiple metal elements in the gut exhibit age‐related declines, suggesting they may function synergistically within the intestinal microenvironment to regulate gut aging processes. Although each metal element likely exerts distinct mechanistic effects, such as Mg's role in regulating PTMs or other metal elements' potential involvement in enzymatic reactions or oxidative stress responses, their coordinated decline appears to disrupt the delicate balance required for gut homeostasis (Einhorn et al. 2024). This observation points to a complex network of metal element‐dependent pathways that together maintain barrier integrity, where combined depletion of multiple metal elements with age may have more profound consequences than the deficiency of any single metal element (Morel et al. 2022). Elucidating these interaction networks in future studies may provide a foundation for multi‐targeted nutritional interventions designed to restore metal element balance and thereby more effectively prevent gut aging and related pathologies.

## 4 Methods

### 4.1 Ethics Statement

This study complied with ethical guidelines for the care and use of animals. All procedures involving non‐human primates were approved by the Institutional Animal Care and Use Committee of Yuanxi Biotech Inc. in Guangzhou (approval no. YXSW‐2016‐01). Human distal normal gut tissue collection from cancer patients and use were authorized by the Ethics Committee of Biology Research at West China Hospital, Sichuan University (approval no. 2020(374)), with informed consent obtained from participants or their families. The use and care of mice were approved by the Animal Experiment Ethics Committee of the State Key Laboratory of Biotherapy at Sichuan University (approval no. 20220531065).

### 4.2 Animal Models and Human Tissue Acquisition

The *Macaca fascicularis* were maintained under controlled laboratory conditions in Guangzhou, China, with an ambient temperature of 25°C and a 12‐h light/dark cycle. Prior to inclusion, all animals were confirmed to be free of clinical symptoms and without prior experimental exposure that could influence normal aging or predispose them to disease. Colon tissues were collected from 6 young (3–4 years old; 3 males and 3 females), 6 middle‐aged (9–10 years old; 3 males and 3 females), and 6 aged (15–16 years old; 3 males and 3 females) monkeys. All samples were immediately flash‐frozen in liquid nitrogen. C57BL/6J mice were obtained from Beijing HFK Bioscience Co. Ltd. (Beijing, China) and housed in a specific pathogen‐free (SPF) facility under a 12‐h light/dark cycle. Before experiments, mice were group‐housed with free access to a standard chow diet and water. Environmental conditions were kept consistent across both experimental and control groups. For the human cohort, with approval from the Research Ethics Committee (approval no. 2020(374)), distal normal tissues (DNTs; 10–15 cm from the tumor margin) were collected from 76 colorectal cancer (CRC) patients enrolled in our previous study (Zhang et al. 2024). All human tissue samples were promptly snap‐frozen in liquid nitrogen and stored at −80°C for long‐term preservation.

### 4.3 Proteomic, Phosphoproteomic, and *N*‐Glycoproteomic Analysis

For proteomic analysis, we applied a strategy similar to that described previously (Zhang et al. 2023). Proteins were extracted from human DNTs, monkey colon tissues or mouse colon tissues. The extracts were digested and labeled with tandem mass tag (TMT) reagents (Thermo Fisher Scientific, 90110 or A37724). TMT‐labeled peptides were desalted, fractionated into 120 fractions by basic reverse‐phase HPLC, and then combined into 20 fractions. The peptides were desalted and analyzed on a Q Exactive Plus coupled with an EASY‐nLC 1000 system (Thermo Fisher Scientific). Peptides were separated on a C18 column (75 μm × 25 cm, DIKMA) using a 90‐min linear gradient (14%–100% buffer B, 80% acetonitrile, 0.1% formic acid) at a flow rate of 330 nL/min. Full MS scans were acquired at a resolution of 70,000 (350–1600 *m*/*z*, automatic gain control (AGC) 3e^6^), and the top 20 precursors were selected for fragmentation (normalized collision energy [NCE] 30%, charge states of *z* = 1 or 8 or unassigned charge states were excluded).

For phosphoproteomics analysis, TMT‐labeled peptides were fractionated on a C18 solid‐phase extraction (SPE) columns (100 mg/1 mL, Waters, WAT023590), and combined into 3 (monkey colon tissues) or 5 fractions (mouse colon tissues). Phosphopeptides were enriched using Fe‐NTA agarose beads (Cube Biotech, 31403‐Fe), desalted and analyzed on either a Q Exactive Plus coupled with an EASY‐nLC 1000 system, a Q Exactive HF‐X coupled with EASY‐nLC 1200 system (Thermo Fisher Scientific), or an Orbitrap Exploris 480 coupled with EASY‐nLC 1200 system (Thermo Fisher Scientific). For the Q Exactive Plus, instrument parameters were set identical to those used in proteomics, except that stepped NCEs (25%, 31%) were applied. For analyses performed on the Q Exactive HF‐X, samples were separated with a 90‐min linear gradient (11%–100% buffer B, 80% acetonitrile, 0.1% formic acid), and full MS scans were acquired at a resolution of 60,000. For the Orbitrap Exploris 480, samples were loaded onto an in‐house pulled and packed analytical column (75 μm × 30 cm, packed with C18 particles) and separated with a 90‐min linear gradient (8%–100% buffer B; 80% acetonitrile, 0.1% formic acid) at a flow rate of 300 nL/min, using stepped NCEs (25%, 31%). Full MS scans were acquired at a resolution of 60,000 (350–1800 *m*/*z*, AGC 75%).

For *N*‐glycoproteomic analysis, a sequential enrichment strategy was applied. After enriching phosphopeptide, the remaining peptides were dried for *N*‐Glycopeptide enrichment according to our previously published protocol (Xu et al. 2023). For monkey colon tissues, after desalting with a C18 ZipTip, peptides were vacuum‐dried, reconstituted in buffer A (2% acetonitrile, 0.1% formic acid) and analyzed on a Q Exactive HF‐X mass spectrometer coupled to a Nano EASY‐nLC 1200 system. Peptides were loaded onto a trap column (100 μm inner diameter × 2.5 cm length) and an analytical column (75 μm inner diameter × 25 cm length), both packed in‐house with C18 particles (DIKMA). Peptide separation was achieved using a 90‐min linear gradient ranging from 11% to 100% buffer B at a flow rate of 330 nL/min. Full MS scans were acquired over a mass range of 700–2000 *m*/*z* with a resolution of 60,000, and the AGC for full MS was set to 3e^6^. The top 20 most intense precursor ions were selected for fragmentation using an isolation window of 0.6 *m*/*z* and fragmentation using stepped NCEs of 20%, 27%, and 31%. Precursor ions with charge states of *z* = 1, 6–8, or with unassigned charge states were excluded. For mouse colon tissues, peptides were analyzed on an Orbitrap Exploris 480 mass spectrometer coupled to an EASY‐nLC 1200 system, using an in‐house packed 30 cm C18 analytical column. Peptide separation was performed with a 90‐min gradient (6%–100% buffer B, 300 nL/min). Full MS scans were acquired at a resolution of 60,000 (350–2000 *m*/*z*, AGC 75%), and the top 20 precursors were fragmented under the same conditions as above.

### 4.4 MS Raw Data Database Searching

Raw files from proteomic and phosphoproteomic analyses were processed using MaxQuant (version 1.6). Spectra from monkey colon samples were searched against a custom *Macaca fascicularis* database containing 76,242 entries, whereas spectra from mouse colon samples were searched against the Swiss‐Prot mouse database (July 2019; 17,019 entries). The precursor ion mass tolerance was set to < 10 ppm, and the fragment ion mass tolerance was set to 0.02 Da. Carbamidomethylation of cysteine was specified as a fixed modification, whereas methionine oxidation and protein *N*‐terminal acetylation were set as variable modifications. Both peptide‐ and protein‐level false discovery rates (FDRs) were controlled at < 1%. The minimum peptide length was six amino acids, and the maximum peptide mass was 12,000 Da. For phosphoproteomic analysis, phosphorylation of serine, threonine, and tyrosine (S/T/Y) residues was additionally defined as a variable modification.

Intact *N*‐glycopeptides searches were performed using GPSeekerPro against a theoretical *N*‐glycan database comprising 108,921 polysaccharide sequence structures, constructed based on known biosynthetic rules and reverse‐synthesis strategies. The protein sequence database was the same as that used for proteomic analysis. Detailed search parameters set are provided in our previous study (Xu et al. 2023).

### 4.5 Data Cleaning

For both proteomics and phosphoproteomics, data preprocessing was performed following the methods as described in our previous study (Zhang et al. 2024). Values recorded as zero were treated as missing and replaced with “NA.” Only proteins and phosphopeptides detected in more than 50% of samples were retained for downstream analysis. For *N*‐glycoproteomics, raw files generated by GPSeeker provided detailed information on peptides, glycosylation sites, glycan compositions, structures, and suggested glycosidic linkages. To minimize batch effects, the total *N*‐glycosylation intensity within each batch was normalized to the same level and defined as the adjusted intensity. For each sample, the adjusted abundance of each site was divided by the corresponding intensity of a common reference sample to generate sample‐to‐reference (S/R) values. For *N*‐glycosylation sites located at the same position and carrying identical glycan compositions but differing in glycosidic linkages, the site with the fewest missing values was retained. Sites with more than 50% missing values across samples were excluded, and the remaining data were log_2_‐transformed prior to downstream analysis.

### 4.6 Bioinformatics Analysis

Cleaned proteomics, phosphoproteomics, and *N*‐glycoproteomics data were analyzed for differential abundance across the three age groups using one‐way ANOVA, and the differences between any two groups were tested by two‐sided Student's *t*‐test. Significance was determined to be *p* < 0.05, and fold change (FC) was calculated as the median log_2_(FC). Spearman's rank correlation was used to calculate the relationship between different omics datasets. Pathway enrichment analysis was performed using DAVID (https://david.ncifcrf.gov/) and Metascape (https://metascape.org/) databases.

### 4.7 Measurement of Metal Content in Tissues Samples

Metal content (Mg, K, Ca, Zn, Rb, Cu, Fe, Mn, and Al) in colon tissues was measured using an Agilent 7700 Series ICP‐MS system operated under standard multi‐element conditions with a helium collision/reaction cell. Detailed analytical procedures are provided in our previous study (Zhang et al. 2024).

### 4.8 Mg‐Deficient Mouse Model

C57BL/6 female mice (8 weeks old and 17 months) were acclimated for 7 days prior to the start of treatment. Within each age group, mice were randomly assigned to receive either a standard chow diet (approximately 500 mg/kg Mg) or a Mg‐deficient chow diet (approximately 80 mg/kg Mg). Both diets were obtained from Beijing HFK Bioscience Co. Ltd. (Beijing, China), and the Mg content was determined based on measured elemental concentrations by ICP‐MS. After 2 months of dietary restriction, mice were sacrificed, and their colons were carefully dissected. Colon length was measured for statistical analysis to assess the effects of Mg deficiency.

### 4.9 Mg‐Treated Mouse Model

C57BL/6 female mice (17 months) were acclimated for 7 days before the start of treatment and randomly assigned to three groups. Within each age group, mice were maintained on a standard chow diet, whereas the Mg‐low and Mg‐high groups were further supplemented with 3 g/L and 10 g/L of MgCl_2_ in sterile drinking water, respectively. The baseline elemental Mg concentration in drinking water was approximately 0.068 mg/L, as determined by ICP‐MS. After 2 months of dietary restriction, mice were sacrificed, and their colons were carefully dissected. Colon length was measured for statistical analysis to assess the effects of Mg‐treated.

### 4.10 Natural Aging Mouse Model

C57BL/6 female mice (4 weeks old and 18 months) were fed with a standard chow diet. After two months, the mice were sacrificed, and their colons were carefully dissected.

### 4.11 DSS‐Induced Colitis Mouse Model Treated With Mg

C57BL/6 male mice (8 weeks old) were acclimated for 7 days before the start of treatment and randomly assigned to six groups. The Normal and DSS groups were maintained on a standard chow diet, whereas the Mg deficiency group received a Mg‐deficient chow diet. The Mg‐low, Mg‐mid, and Mg‐high groups receiving a Mg‐deficient chow diet were further supplemented with 3, 7, and 10 g/L of MgCl_2_ in sterile drinking water, respectively. The baseline elemental Mg concentration in drinking water was approximately 0.068 mg/L, as determined by ICP‐MS. During the colitis induction phase (Day 0 to Day 7), the Normal group was given regular drinking water, whereas the remaining five groups received 3% DSS in drinking water. Following DSS withdrawal, mice continued their respective MgCl_2_ treatments for an additional 3 days before sacrifice. Body weight and disease activity index (DAI) were recorded daily. At the endpoint, mice were euthanized by cervical dislocation, and colons were collected and measured to assess inflammation severity.

### 4.12 Colon Dissection and Tissue Collection

In mice, the colon was defined as the intestinal segment extending from the distal end of the cecum to approximately 0.5 cm proximal to the anus, thereby excluding the rectum. To ensure anatomical consistency and sampling precision across animals, 0.5 cm segments immediately distal to the cecum and adjacent to the rectum at the distal colon were excluded from regional subdivision. The remaining colonic segment was evenly divided into three equal‐length regions, which were operationally defined as the ascending, transverse, and descending colon. For omics analyses, the ascending and descending colon were each further subdivided into two equal portions. Specifically, the segment adjacent to the cecum in the ascending colon and the segment adjacent to the anus in the descending colon were collected for proteomic, phosphoproteomic, and *N*‐glycoproteomic analyses. The remaining colonic tissues were snap‐frozen in liquid nitrogen and stored at −80°C for subsequent experiments.

### 4.13 Cell Experiments

The human normal colonic epithelial cell line NCM460 was obtained from the Cell Bank/Stem Cell Bank, Chinese Academy of Sciences. NCM460 cells were cultured in RPMI 1640 medium (Gibco, 10270‐106), supplemented with 10% (v/v) fetal bovine serum (Gibco, A5669701), 100 U/mL penicillin, and 100 μg/mL streptomycin (Gibco, 15140‐122). The cells were maintained in a humidified incubator at 37°C with 5% CO_2_. To generate TJP2‐Flag‐tagged plasmid, the cDNA encoding TJP2 was cloned into the pLV3‐3 × Flag vector. For TJP2 knockdown, the PLKO.1 vector was used. To establish cell lines overexpressing wild‐type TJP2, the TJP2^S986A^ mutant, and the TJP2^S986D^ mutant, a co‐transfection strategy was employed in HEK293T cells. Specifically, pSPAX2, pMD2.G, pLV3‐3xFlag‐TJP2^WT^, pLV3‐3xFlag‐TJP2^S986A^, pLV3‐3xFlag‐TJP2^S986D^ and corresponding control plasmids were co‐transfected into HEK293T cells. After 48 h, virus‐containing supernatants were harvested and filtered through a 0.22‐μm membrane. To enhance infection efficiency, polybrene (10 μg/mL; Sigma‐Aldrich, S2667) was added to the medium. Target cells were then infected and selected with puromycin (1 μg/mL; Beyotime, ST551) for 48 h.

The shRNA primer sequence targeting TJP2 was: 5′‐CCGGCCTACCTTTGGGCGGTCTATACTCGAGTATAGACCGCCCAAAGGTAGGTTTTTG‐3′.

To construct the TJP2 mutants, 2 paired primers were used and designed as follows:

TJP2^S986A^‐sense: 5′‐TTAGGGACAATGCCCCGCCCCCAGC‐3′.

TJP2^S986A^‐antisense: 5′‐GCTGGGGGCGGGGCATTGTCCCTAA‐3′.

TJP2^S986D^‐sense: 5′‐TTAGGGACAATGACCCGCCCCCAGC‐3′.

TJP2^S986D^‐antisense: 5′‐GCTGGGGGCGGGTCATTGTCCCTAA‐3′.

### 4.14 Western Blotting Analysis

Proteins were extracted from colon tissues and cultured cells using RIPA buffer (150 mM NaCl, 50 mM Tris (pH 7.5), 1% (v/v) NP‐40, 0.5% (w/v) sodium deoxycholate) supplemented with protease and phosphatase inhibitor cocktails. Protein concentrations were determined using the Bicinchoninic Acid (BCA) assay. Equal amounts of protein were separated by 10% SDS‐PAGE and transferred onto PVDF membranes. Membranes were blocked with 5% nonfat dry milk in PBST (137 mM NaCl, 2.7 mM KCl, 10 mM Na_2_HPO_4_, 1.8 mM KH_2_PO_4_, 0.1% (w/v) Tween 20 detergent) and incubated overnight at 4°C with primary antibodies against CTNNA1 (HuaBio, ER62912; 1:1000), CTNNB1 (HuaBio, ET1601‐5; 1:1000), CTNND1 (HuaBio, ER1803‐71; 1:1000), CLDN1 (HuaBio, ER1906‐37; 1:1000), P21 (Proteintech, 10355‐1‐AP; 1:1000), TJP2 (Proteintech, 18900‐1‐AP; 1:1000), JAM1 (HuaBio, ET1610‐90; 1:1000) and GAPDH (Proteintech, 60004‐1‐Ig; 1:5000). Following three PBST washes, membranes were incubated with appropriate HRP‐conjugated secondary antibodies at room temperature for 1 h. Protein bands were visualized using Immobilon Western HRP Substrate (Millipore) or an equivalent chemiluminescent detection system.

### 4.15 Histopathology

Colon tissues from mice were fixed in 4% paraformaldehyde and subsequently embedded in paraffin. The embedded samples were sliced into thin sections at a thickness of 5 μm and stained using hematoxylin and eosin (H&E). Slides were scanned with an automated digital slide scanner (PANNORAMIC MIDI, 3DHISTECH) and analyzed using CaseViewer software (RRID: SCR_017654). Histological evaluation was conducted independently by two blinded observers using previously established scoring criteria (Wang et al. 2024).

### 4.16 UK Biobank Data Analysis

Study population and design. The UK Biobank is a large, prospective cohort study that recruited over 500,000 participants aged 37–73 years from 2006 to 2010. Each participant completed self‐reported touchscreen questionnaires and verbal interviews, underwent physical measurements, and provided biological samples at one of 22 assessment centers in England, Wales, and Scotland (Collins 2012). For this study, the sample was initially restricted to 210,863 participants with complete data on dietary Mg intake. Participants with prevalent gut disorders at baseline (*n* = 26,436), or with abnormal energy intake of < 800 or > 4200 kcal/day for males and < 600 or > 3500 kcal/day for females (*n* = 2214) were excluded, leaving 182,213 participants for the main analyses.

Assessment of dietary Mg intake. Dietary Mg intake information was collected by using the web‐based, self‐administered 24‐h recall questionnaire “Oxford WebQ” in 2009. More details of the Oxford WebQ have been described previously (Liu et al. 2011). Energy adjustment for Mg intake was performed using the residual method (McCullough and Byrd 2023).

Ascertainment of outcomes. Health outcomes of participants in the UK Biobank were obtained through linkage to electronic medical records, updated regularly. The primary outcomes included five common digestive diseases, defined using codes of the International Classification of Diseases 9th Revision (ICD‐9) and ICD‐10: Crohn's disease, ulcerative colitis, irritable bowel syndrome, diverticular disease, and celiac disease.

### 4.17 Statistical Analysis

Baseline characteristics were described according to dietary Mg intake (sufficient vs. deficient). Data were summarized as mean ± standard deviation (SD) for normally distributed continuous variables, median [interquartile range, IQR] for nonnormally distributed continuous variables, and frequency (percentage) for categorical variables. Follow‐up duration was defined as the time from baseline to the first diagnosis of outcomes, death, or the censoring (31 October 2022), whichever occurred first. The associations between levels of total Mg and the risks of intestinal diseases were assessed using multivariate Cox proportional hazards regression to estimate the hazard ratios (HRs) and 95% confidence interval (CI), adjusting for age, sex (female, male), ethnicity (whites, others), total energy intake (in tertiles, specified by sex), physical activity, smoking status, alcohol consumption, and body mass index (BMI, underweight/normal, overweight, obesity).

## Author Contributions

L.D. designed the project, supervised the experiments, and wrote the paper. R.Z., M.H., W.L., S.H., Y.L., and B.C. participated in all the experiments and mass spectrometry data analysis. P.L. helped to perform the ICP‐MS analysis of metal elements. Z.L., Y.Z., J.W., J.Y., and M.G. involved in the analysis of UK Biobank Data. H.X., Y.P., and H.‐N.C. helped to collect the human and monkey samples and provided helpful suggestions for the bioinformatics analysis.

## Funding

This work was supported by National Key R&D Program of China (2022YFA1303200), Noncommunicable Chronic Diseases‐National Science and Technology Major Project (2024ZD0531100), Science and Technology Project of Sichuan Province (2024YFFK0099, 2026NSFSC0924), National Clinical Research Center for Geriatrics of West China Hospital (Z2024JC002), West China Hospital 1.3.5 project for disciplines of excellence (ZYYC23013), China Postdoctoral Science Foundation (2024M762204), Postdoctoral Research Fund of West China Hospital (2024HXBH025), Yang Peng Mercantile Limited (ZL), Zhejiang Key Laboratory of Intelligent Preventive Medicine, and National Natural Science Foundation of China (82401845).

## Conflicts of Interest

The authors declare no conflicts of interest.

## Supporting information


**Appendix S1:** acel70446‐sup‐0001‐AppendixS1.zip.

**Figure S1:** acel70446‐sup‐0002‐FiguresS1‐S8.pdf.
**Figure S2:** acel70446‐sup‐0002‐FiguresS1‐S8.pdf.
**Figure S3:** acel70446‐sup‐0002‐FiguresS1‐S8.pdf.
**Figure S4:** acel70446‐sup‐0002‐FiguresS1‐S8.pdf.
**Figure S5:** acel70446‐sup‐0002‐FiguresS1‐S8.pdf.
**Figure S6:** acel70446‐sup‐0002‐FiguresS1‐S8.pdf.
**Figure S7:** acel70446‐sup‐0002‐FiguresS1‐S8.pdf.
**Figure S8:** acel70446‐sup‐0002‐FiguresS1‐S8.pdf.

**Table S1:** acel70446‐sup‐0003‐TableS1.xlsx.

## Data Availability

Raw MS proteomic data of *Macaca fascicularis* have been deposited in the ProteomeXchange Consortium via the iProX partner repository (Chen et al. 2022) under the dataset identifier PXD053818. In addition, the phosphoproteomic and *N*‐glycoproteomic datasets of *M. fascicularis* and mice, together with the proteomic data of mice, are available in the ProteomeXchange Consortium with the iProX accession number PXD067494. The data that support the findings of this study are available in UK Biobank at [https://www.ukbiobank.ac.uk/], reference number [61856].
