# Supplementary figures and images for: Magnesium Deficiency Accelerates Gut Aging and Increases Susceptibility to Colitis
Source: Aging Cell. 2026 Mar 16;25(3):e70446. doi: 10.1111/acel.70446 (PMC13093417; doi:10.1111/acel.70446)

Figure 2E

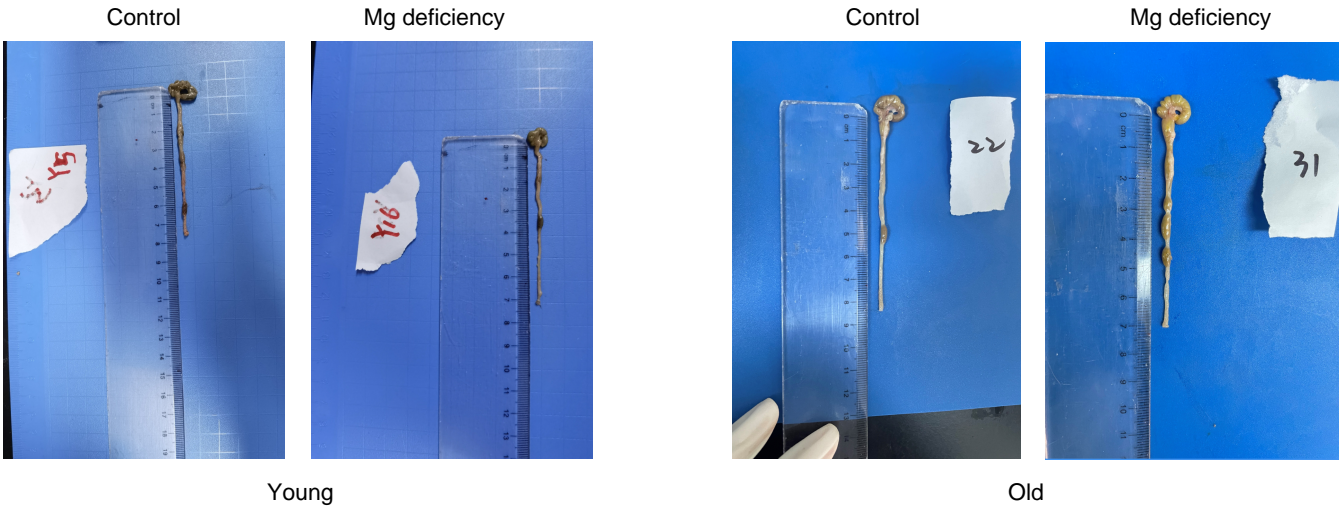

Figure 2F

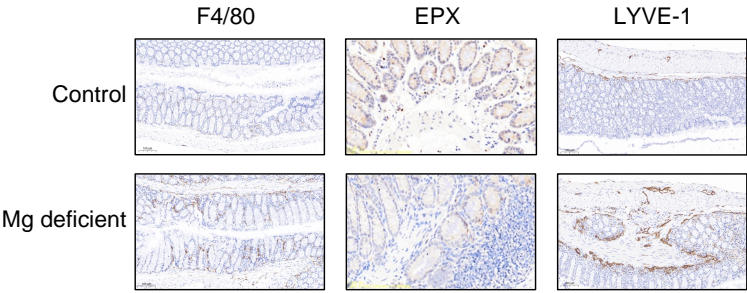

Figure 2I

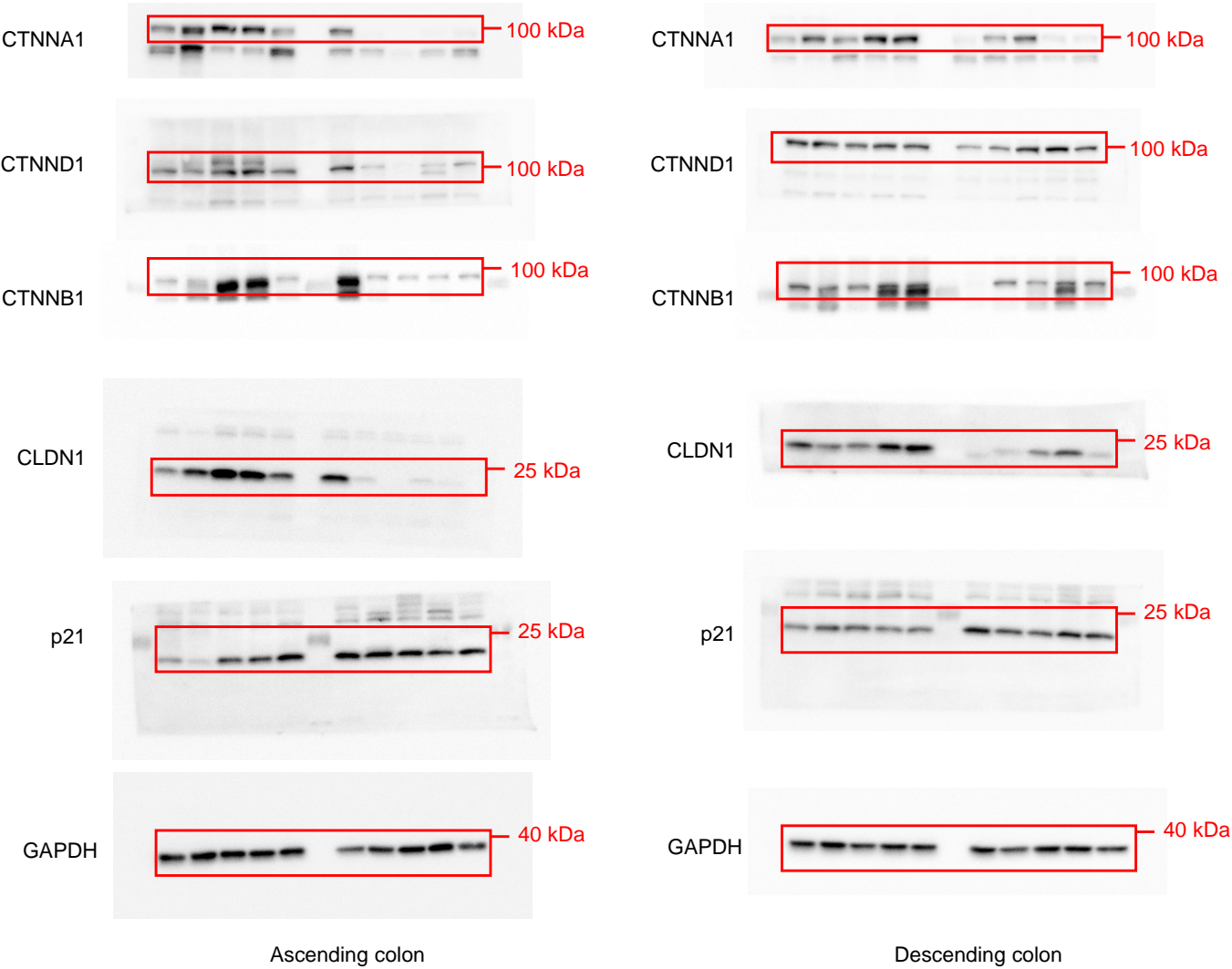

Figure 2L

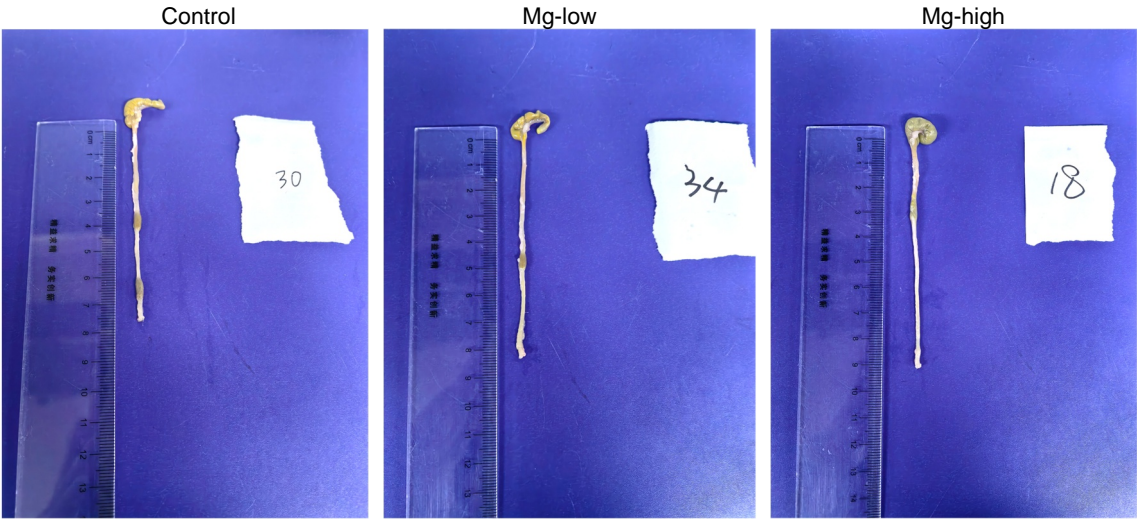

Figure 2N

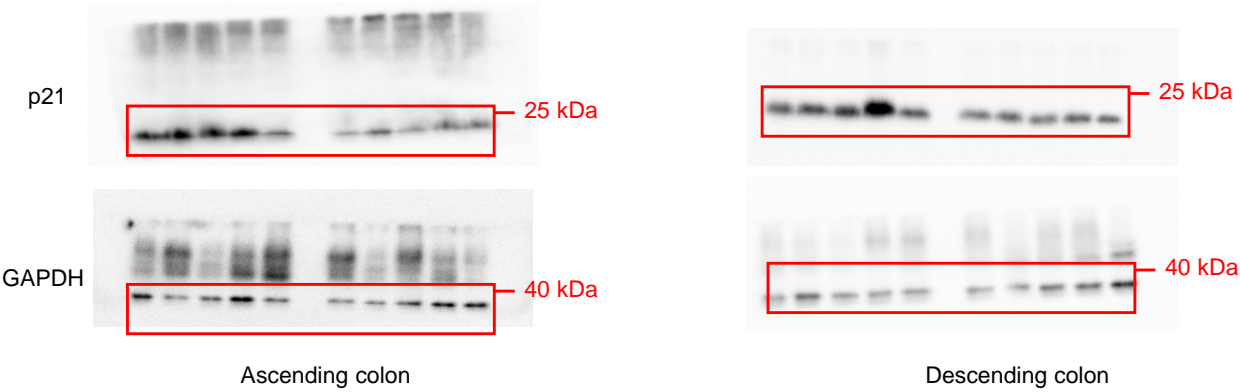

Supplement: Supplementary file 1 — Appendix S1: acel70446‐sup‐0001‐AppendixS1.zip. [file ACEL-25-e70446-s003.zip › Source Image Figure 2.pdf]

Figure 4D

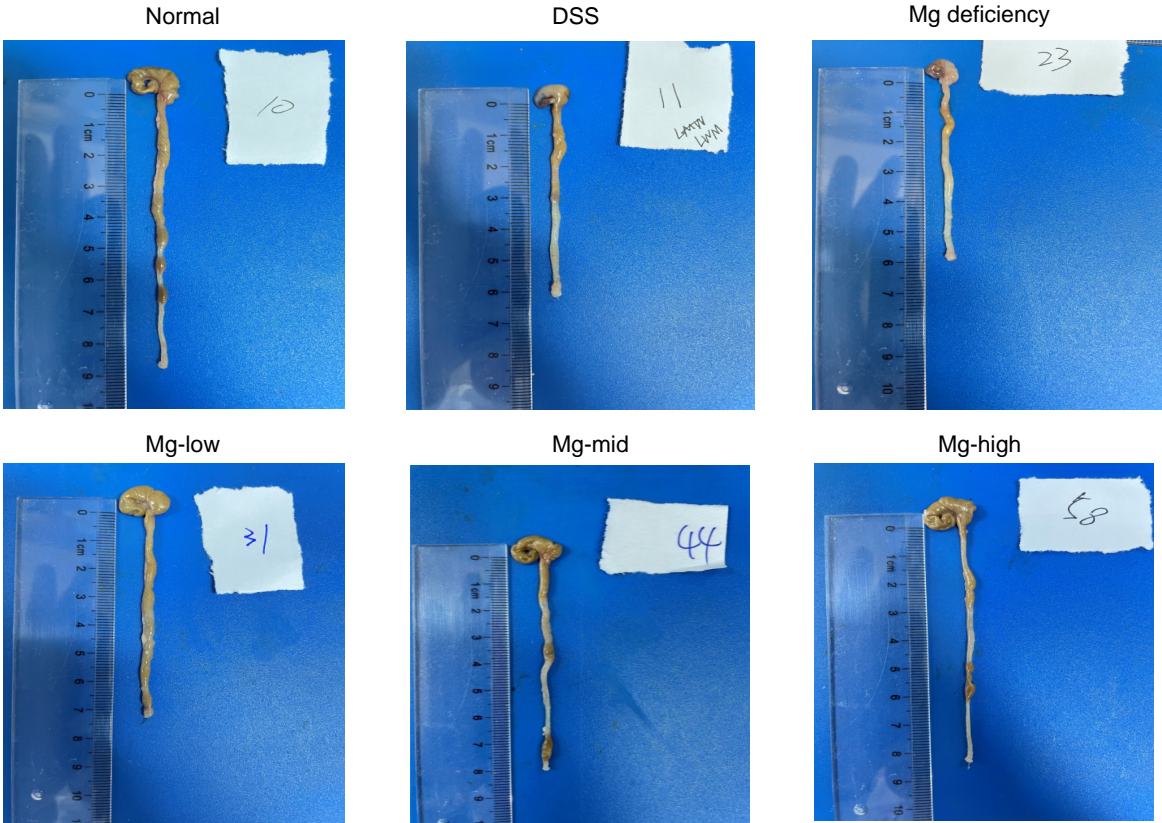

Figure 4E

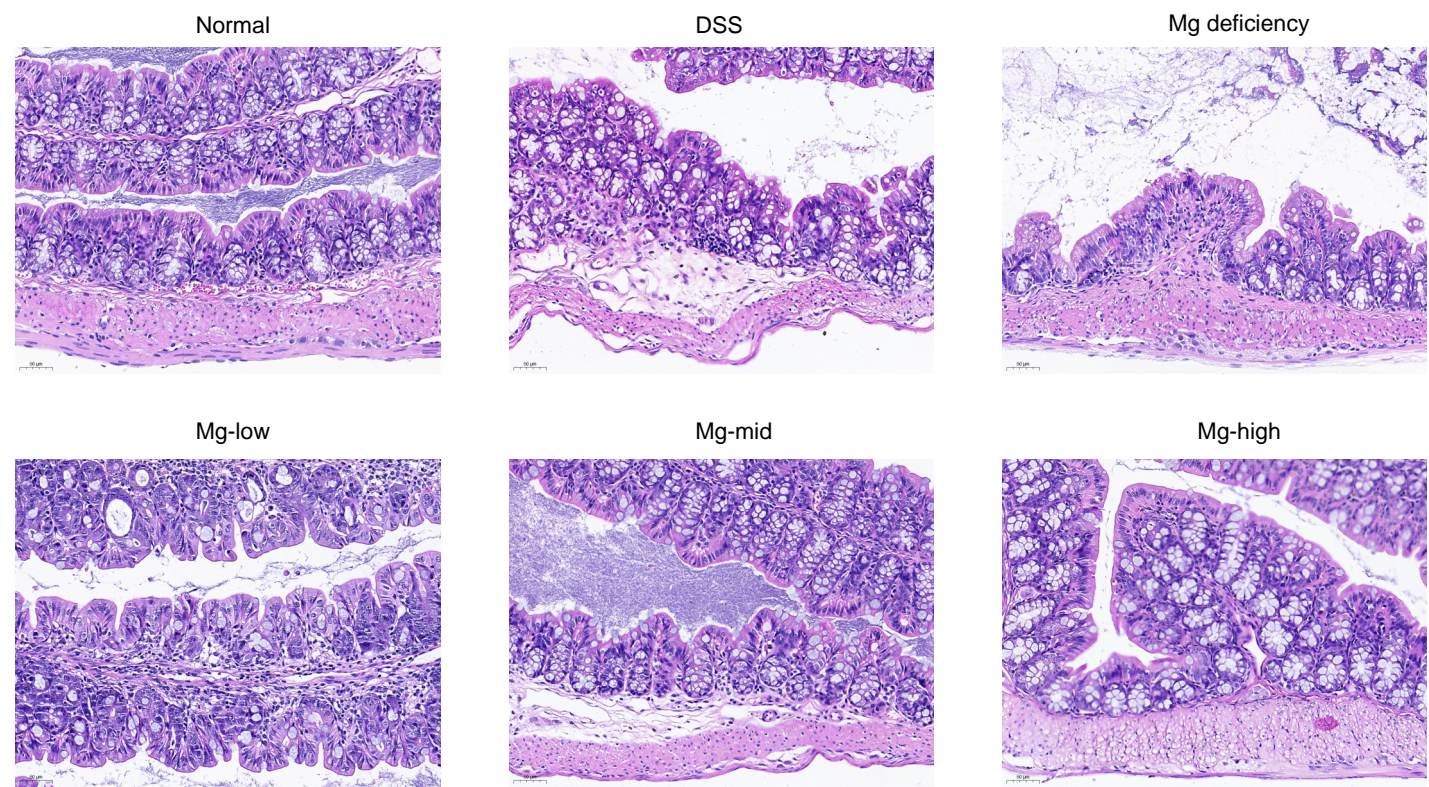

Figure 4G

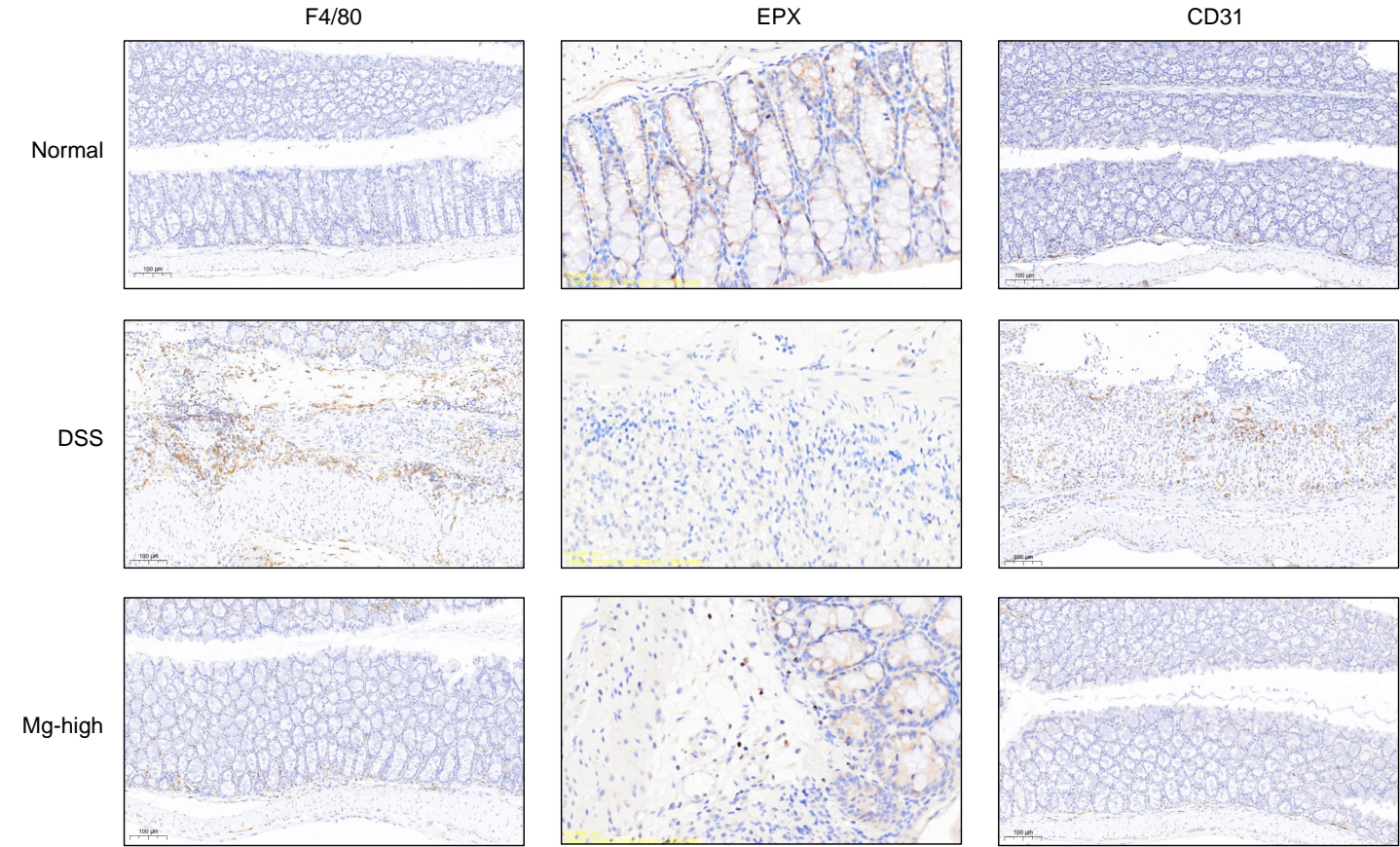

Supplement: Supplementary file 1 — Appendix S1: acel70446‐sup‐0001‐AppendixS1.zip. [file ACEL-25-e70446-s003.zip › Source Image Figure 4.pdf]

Figure 5F

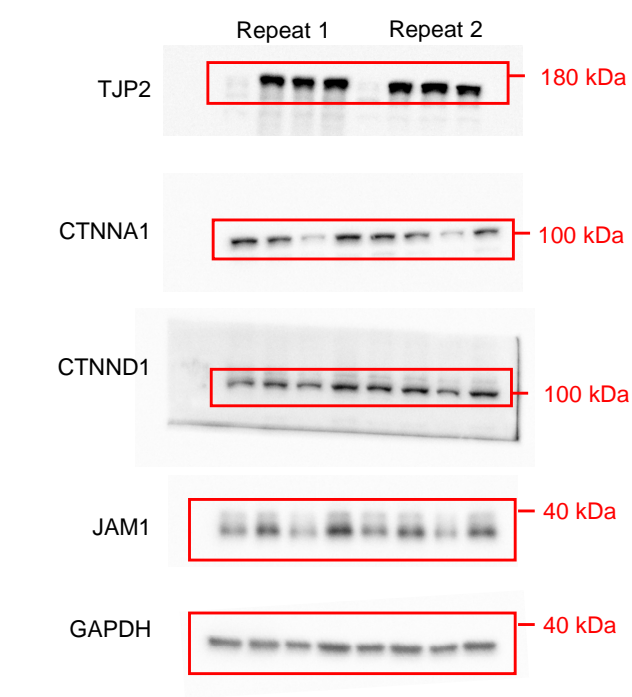

Figure 5G

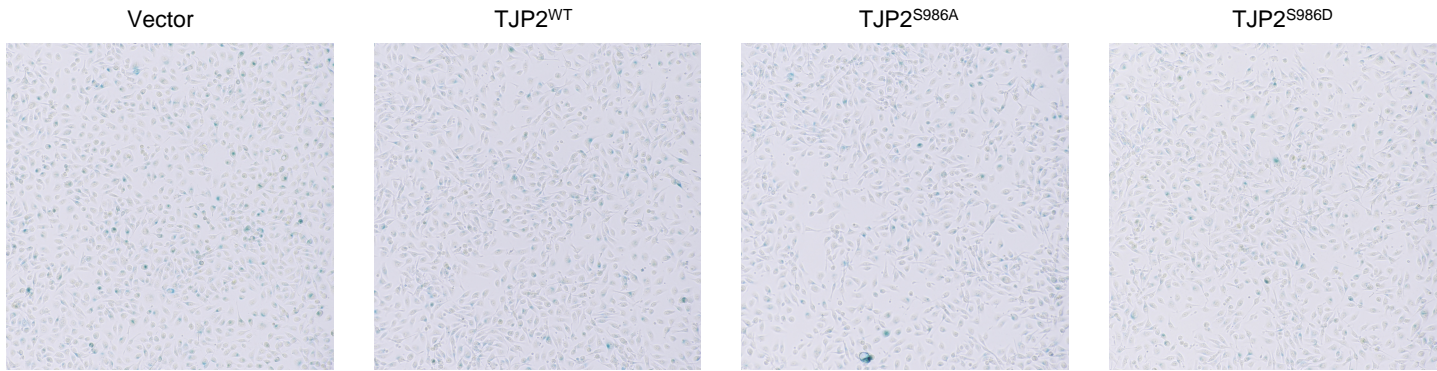

Supplement: Supplementary file 1 — Appendix S1: acel70446‐sup‐0001‐AppendixS1.zip. [file ACEL-25-e70446-s003.zip › Source Image Figure 5.pdf]

Figure S5C

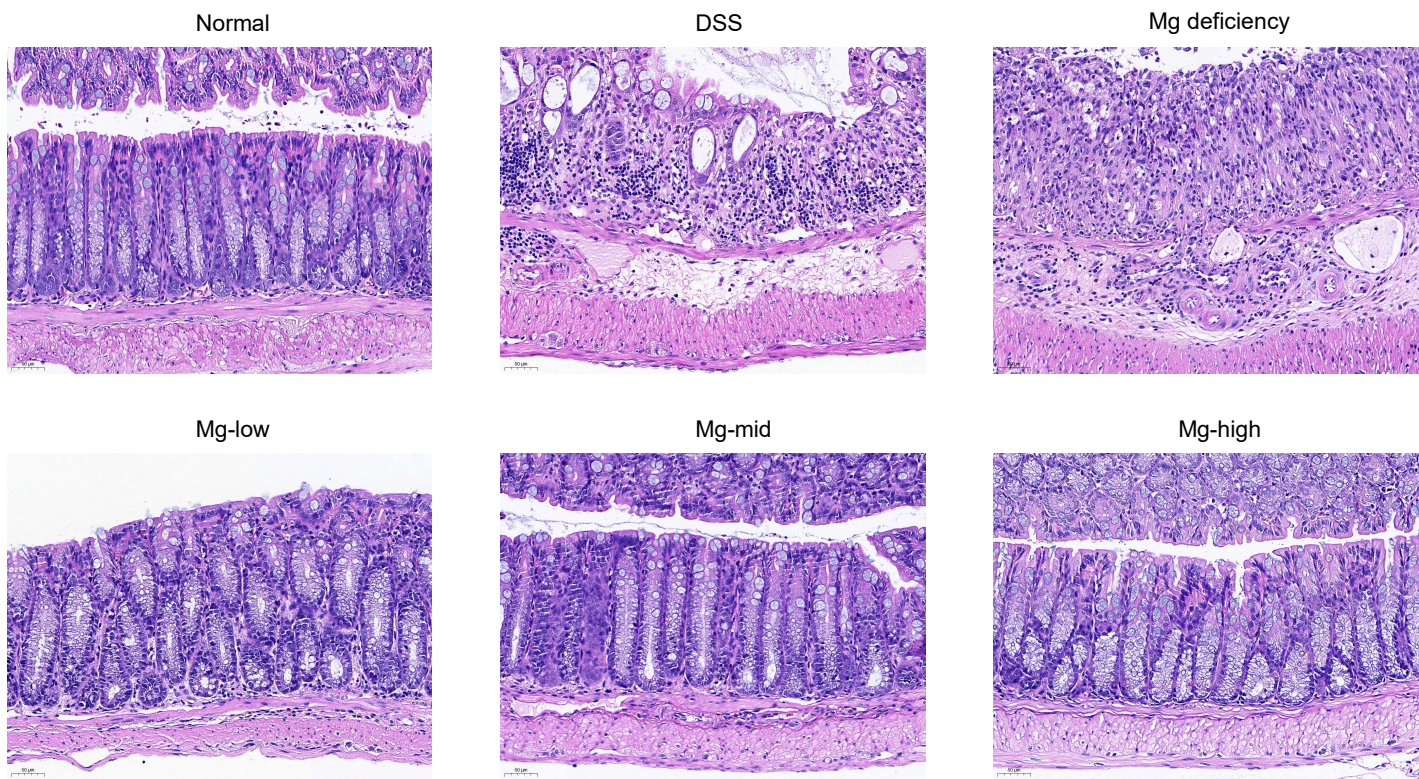

Supplement: Supplementary file 1 — Appendix S1: acel70446‐sup‐0001‐AppendixS1.zip. [file ACEL-25-e70446-s003.zip › Source Image Figure S5.pdf]
