# Supplementary material for: Magnesium Deficiency Accelerates Gut Aging and Increases Susceptibility to Colitis
Source: Aging Cell. 2026 Mar 16;25(3):e70446. doi: 10.1111/acel.70446 (PMC13093417; doi:10.1111/acel.70446)
Supplement: Supplementary file 2 — Figure S1: acel70446‐sup‐0002‐FiguresS1‐S8.pdf. Figure S2: acel70446‐sup‐0002‐FiguresS1‐S8.pdf. Figure S3: acel70446‐sup‐0002‐FiguresS1‐S8.pdf. Figure S4: acel70446‐sup‐0002‐FiguresS1‐S8.pdf. Figure S5: acel70446‐sup‐0002‐FiguresS1‐S8.pdf. Figure S6: acel70446‐sup‐0002‐FiguresS1‐S8.pdf. Figure S7: acel70446‐sup‐0002‐FiguresS1‐S8.pdf. Figure S8: acel70446‐sup‐0002‐FiguresS1‐S8.pdf. [file ACEL-25-e70446-s002.pdf]

Figure S1

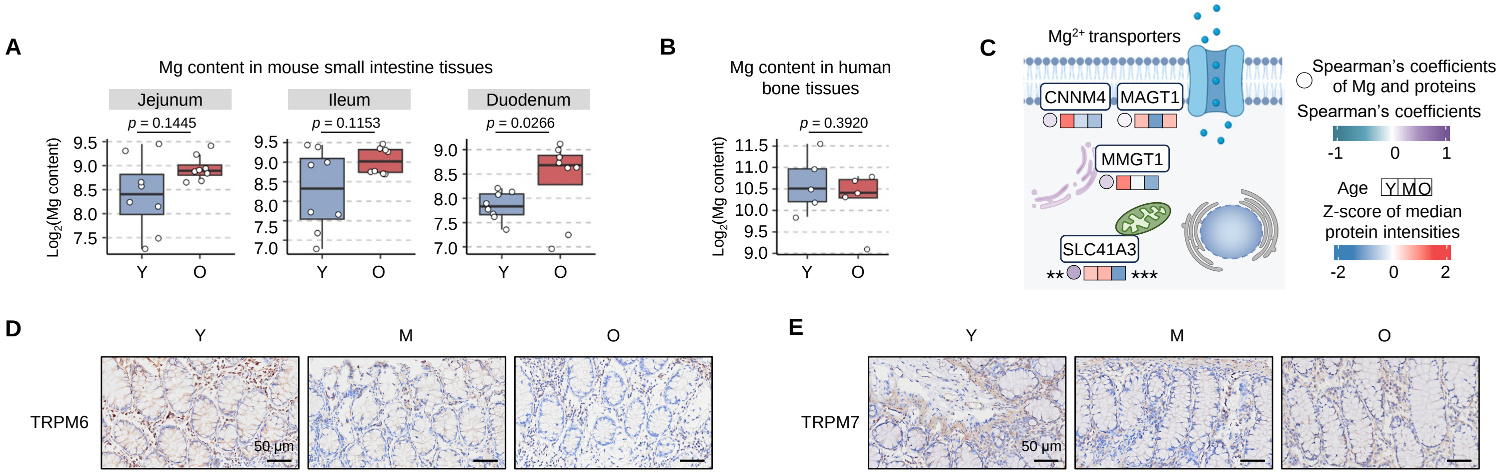

FIGURE S1 Mg content and Mg^{2+} transporter expression.
(A) Mg content in mouse small intestine tissues (two-sided Student's t-test, median ± quartiles). (B) Mg content in human bone tissues (two-sided Student's t-test, median ± quartiles). (C) Scaled expression of Mg^{2+} transporters across three age groups in monkey gut tissues (one-way ANOVA), and Spearman's coefficients of Mg levels and proteins expression. The *p* value is labeled outside the box or circle. *$p < 0.05$, **$p < 0.01$ and ***$p < 0.001$. (D,E) Representative immunohistochemistry images of TRPM6 (D) and TRPM7 (E) expression in monkey gut tissues. Scale bars, 50 μm. Raw and processed data for drawing are provided in Source Data Figure S1.

Figure S2

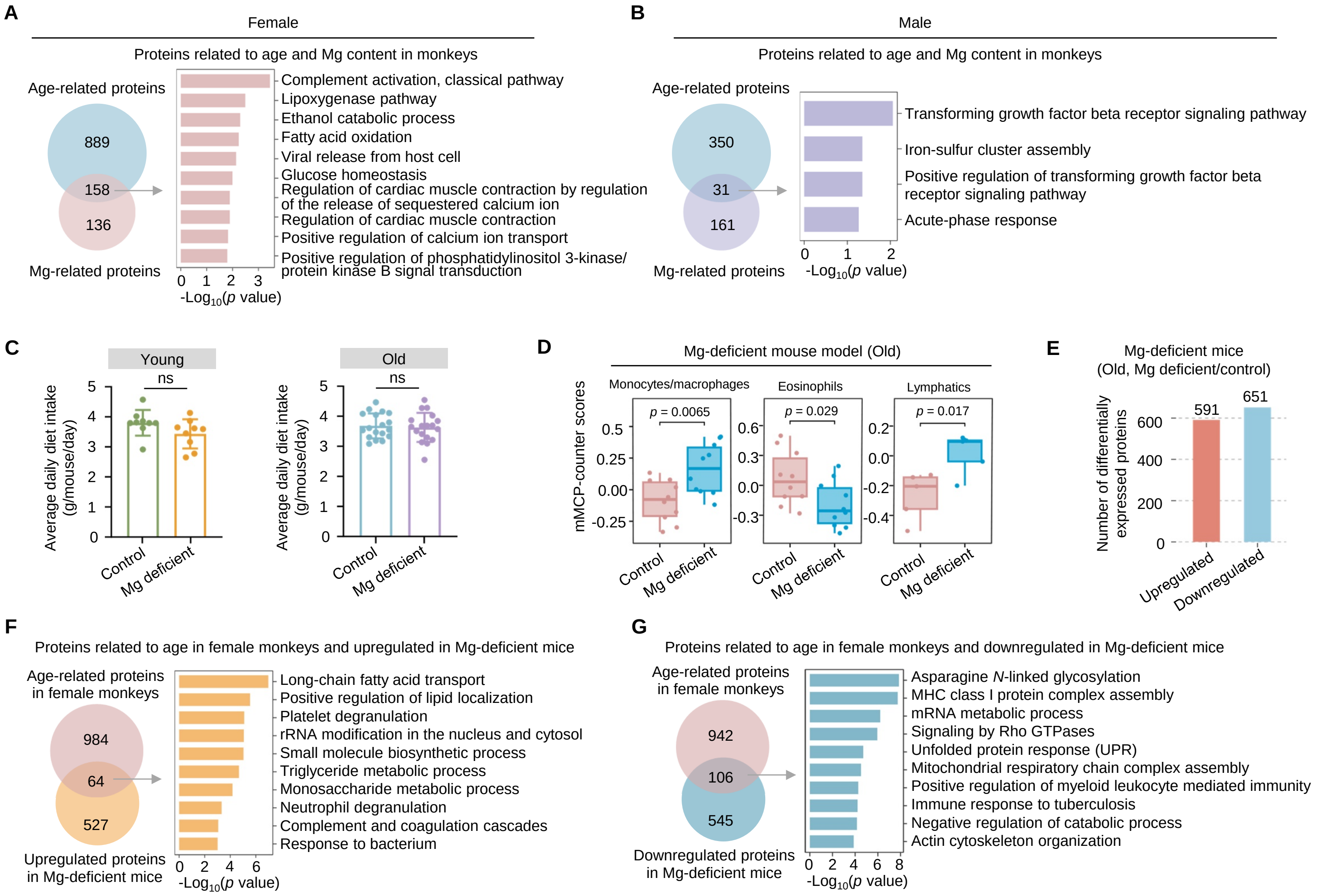

FIGURE S2 Sex-resolved Mg-associated proteome of the large intestine in monkeys. (A,B) Venn diagram displaying overlap between Mg-related (Spearman's correlations, $p < 0.05$) and age-related (one-way ANOVA, $p < 0.05$) proteins in female (A) or male (B) monkeys. Enriched pathways are shown on the right. (C) Box plot displaying average daily diet intake in control and Mg-deficient diets (two-sided Student's t-test, mean ± s.d.). (D) Box plots showing mMCP-counter scores in control and Mg-deficient mice (two-sided Student's t-test, median ± quartiles). (E) Bar plots showing the number of differentially expressed proteins between the control and Mg-deficient groups (two-sided Student's t-test, |log2(fold change)| > 0.263 and P < 0.05). (F,G) Venn diagram displaying overlap between age-related proteins in female monkeys (one-way ANOVA, $p < 0.05$) and proteins upregulated (F) or downregulated (G) in Mg-deficient mice (two-sided Student's t-test, $p < 0.05$). Enriched pathways are shown on the right. Raw and processed data for drawing are provided in Source Data Figure S2.

Figure S3

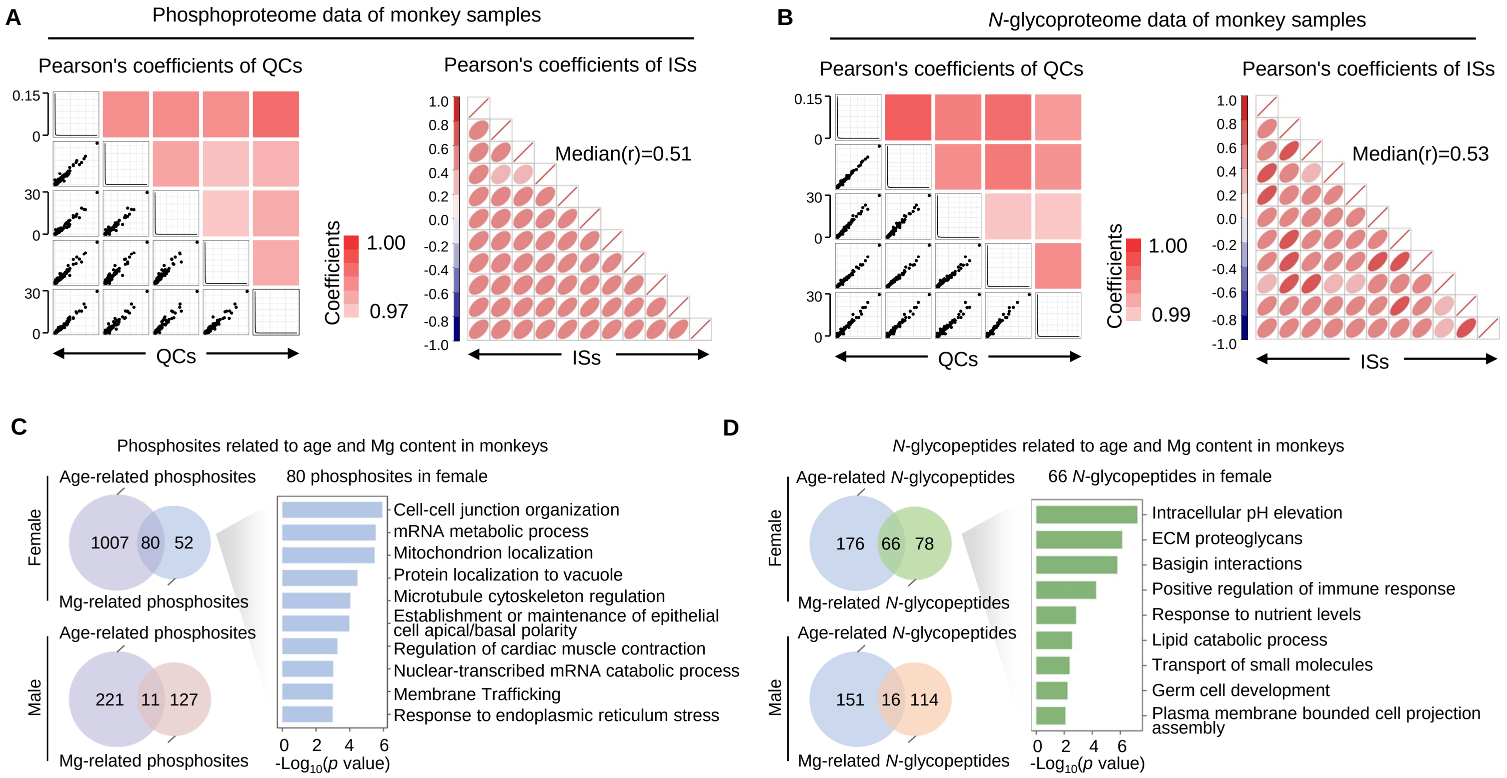

FIGURE S3 Sex-resolved Mg-associated phosphoproteome and *N*-glycoproteome of the large intestine in monkeys.

(A,B) Pearson's correlation analysis of quality control (QC) samples (left) and internal standards (IS) (right) to evaluate the machine stability and the quality of phosphoproteomic (A) and *N*-glycoproteomic (B) data. (C) Venn diagram displaying overlap between Mg-related (Spearman's correlations, p < 0.05) and age-related (one-way ANOVA, p < 0.05) phosphosites in female or male monkeys. The pathways enriched using the corresponding phosphoproteins of 80 overlapping phosphosites in female are shown on the right. (D) Venn diagram displaying overlap between Mg-related (Spearman's correlations, p < 0.05) and age-related (one-way ANOVA, p < 0.05) *N*-glycopeptides in female or male monkeys. The pathways enriched using the corresponding glycoproteins of 66 overlapping *N*-glycopeptides in female are shown on the right. Raw and processed data for drawing are provided in Source Data Figure S3.

Figure S4

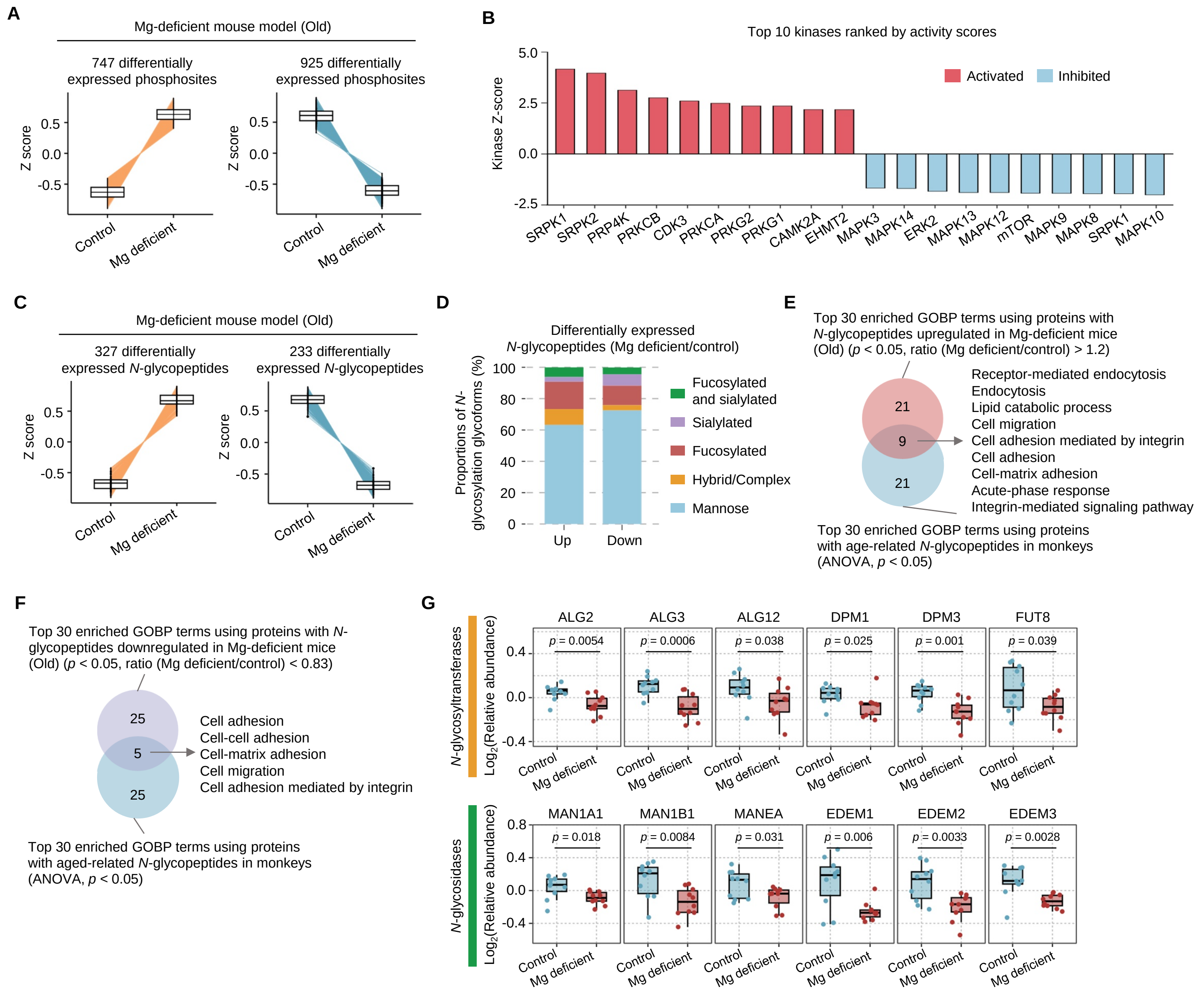

FIGURE S4 Phosphorylation and *N*-glycosylation landscape in Mg-deficient mice.
(A) Expression patterns of upregulated (left) and downregulated (right) phosphosites in Mg-deficient mice (two-sided Student's *t*-test, $p < 0.05$). (B) Kinase-substrate prediction analysis was performed using the KSEAapp. Red represents activated kinase activity, while blue represents inhibited kinase activity. (C) Expression patterns of upregulated (left) and downregulated (right) *N*-glycopeptides in Mg-deficient mice (two-sided Student's *t*-test, $p < 0.05$). (D) Percentage of five glycan types in upregulated and downregulated *N*-glycopeptides of Mg-deficient mice. (E,F) Venn diagram showing overlapping pathways enriched by proteins with *N*-glycopeptides upregulated (E) and downregulated (F) in Mg-deficient mice and proteins with age-related *N*-glycopeptides in monkeys. Overlapping pathways are shown on the right. (G) Box plot displaying the levels of *N*-glycosyltransferases and *N*-glycosidases in control and Mg-deficient groups (two-sided Student's *t*-test, median ± quartiles). Raw and processed data for drawing are provided in Source Data Figure S4.

Figure S5

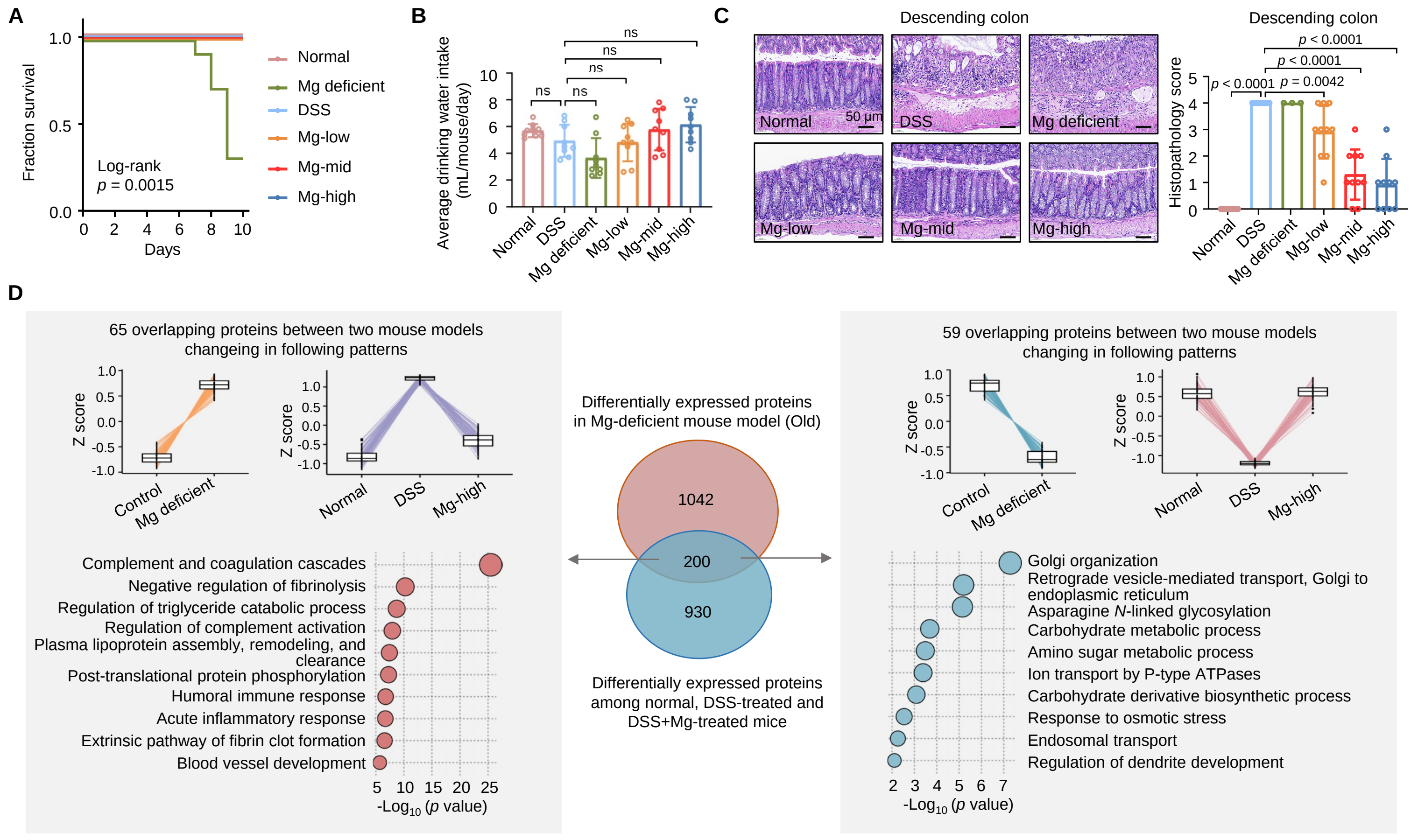

FIGURE S3 Comparative proteomic analysis in Mg-deficient and DSS-induced colitis mice.
(A) Survival analysis of DSS-induced colitis mice (Log-rank test). (B) Box plot illustrates statistical results of average daily drinking water intake per mouse (two-sided Student's t-test, mean ± s.d.). (C) Representative H&E images of descending colons. Scale bars, 50 μm. Box plot illustrates statistical results of histopathology score (0 = none; 1 = very mild; 2 = mild; 3 = moderate; 4 = severe) (two-sided Student's t-test, mean ± s.d.). (D) Venn diagram displaying overlapping proteins between Mg-deficient and DSS-induced colitis mouse models (two-sided Student's *t*-test, $p < 0.05$) (middle). The upper left panel shows expression patterns of proteins upregulated in Mg-deficient mice and in the DSS-induced colitis group but reduced with Mg supplementation, with enriched pathways shown in the lower left panel. The upper right panel shows expression patterns of proteins downregulated in Mg-deficient mice and in the DSS-induced colitis group but increased with Mg supplementation, with enriched pathways shown in the lower right panel. Raw and processed data for drawing are provided in Source Data Figure S5.

Figure S6

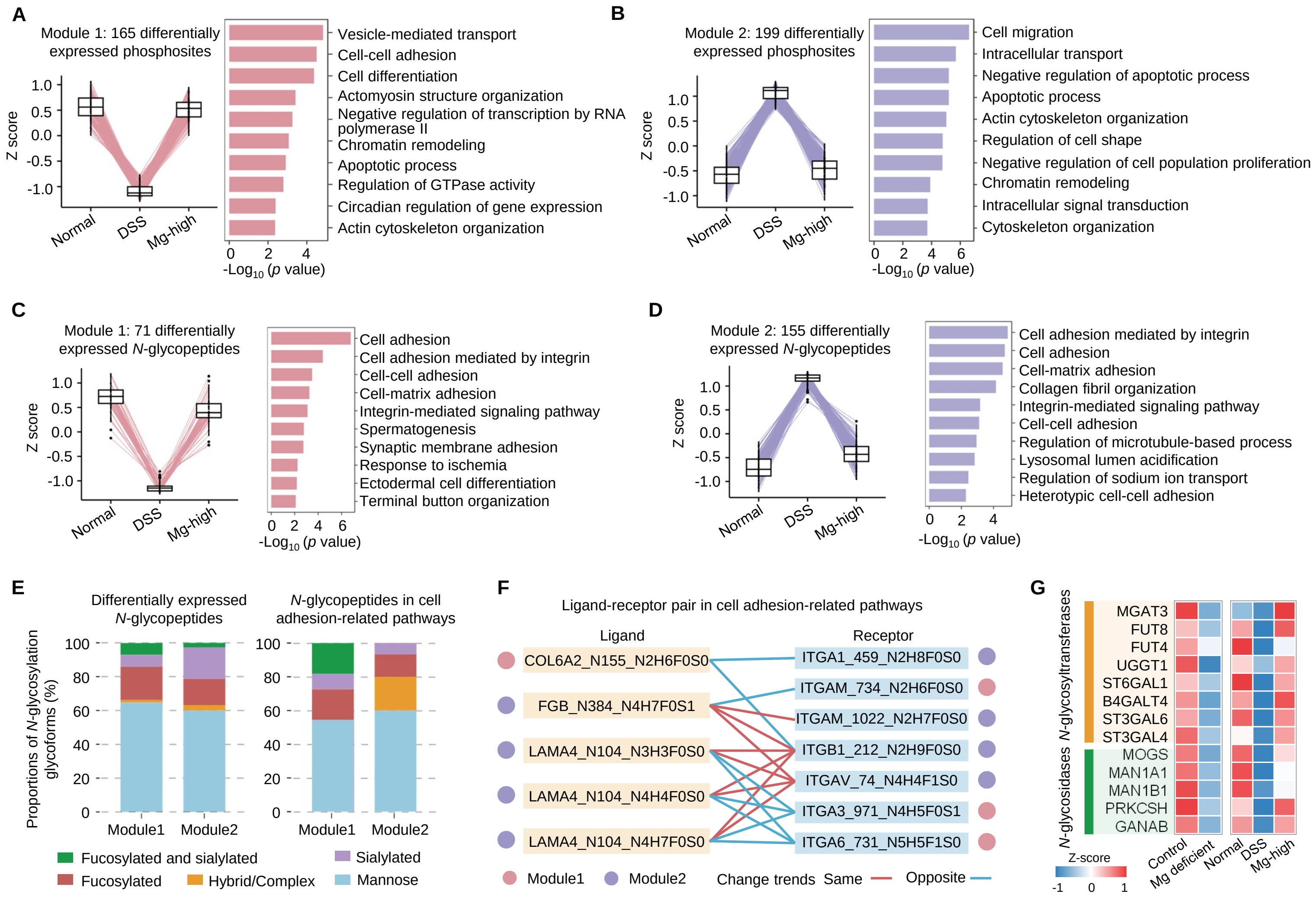

FIGURE S6 Phosphorylation and *N*-glycosylation landscape in DSS-induced colitis mouse model.
(A) Expression patterns of phosphosites downregulated in the DSS-induced colitis group but increased with Mg supplementation are shown in the left panel, with enriched pathways shown in the right panel (two-sided Student's *t*-test, $p < 0.05$). (B) Expression patterns of the phosphosites upregulated in the DSS-induced colitis group but declined with Mg supplementation are shown in the left panel, with enriched pathways shown in the right panel (two-sided Student's *t*-test, $p < 0.05$). (C) Expression patterns of *N*-glycopeptides downregulated in the DSS-induced colitis group but increased with Mg supplementation are shown in the left panel, with enriched pathways shown in the left panel (two-sided Student's *t*-test, $p < 0.05$). (D) Expression patterns of *N*-glycopeptides upregulated in the DSS-induced colitis group but declined with Mg supplementation are shown in the left panel, with enriched pathways shown in the right panel (two-sided Student's *t*-test, $p < 0.05$). (E) Percentage distribution of five glycan types in all differentially expressed *N*-glycopeptides (left) and cell adhesion-related *N*-glycopeptides (left) in DSS-induced colitis mice. (F) Trends of *N*-glycopeptides in ligand-receptor pair in cell adhesion-related pathways. (G) Heat map showing changes of *N*-glycosyltransferases and *N*-glycosidases in Mg-deficient and DSS-induced colitis mice. Raw and processed data for drawing are provided in Source Data Figure S6.

Figure S7

A Sensitivity of Analysis of the associations between daily dietary Mg intake and the risk of five common gut disorders during the 13-year follow-up in the UK Biobank after excluding the under-nutrition subjects (2009-2022, *N* = 139,618)

Diseases	Quintile 1	Quintile 2	*p2*	Quintile 3	*p3*	Quintile 4	*p4*	Quintile 5	*p5*
Crohn disease	1.00 (reference)	0.785 (0.585-1.052)	0.105	0.714 (0.526-0.967)	0.030*	0.624 (0.455-0.856)	0.003*	0.517 (0.371-0.720)	0.016*
Ulcerative colitis	1.00 (reference)	0.780 (0.625-0.974)	0.028*	0.786 (0.629-0.983)	0.034*	0.694 (0.551-0.874)	0.002*	0.651 (0.516-0.822)	<0.001*
Irritable bowel syndrome	1.00 (reference)	0.789 (0.694-0.896)	<0.001*	0.769 (0.676-0.874)	<0.001*	0.749 (0.659-0.852)	<0.001*	0.803 (0.708-0.912)	<0.001*
Diverticular disease	1.00 (reference)	0.933 (0.887-0.983)	0.008*	0.891 (0.846-0.938)	<0.001*	0.842 (0.799-0.887)	<0.001*	0.789 (0.748-0.832)	<0.001*
Celiac disease	1.00 (reference)	1.053 (0.819-1.353)	0.688	1.025 (0.797-1.320)	0.846	0.865 (0.666-1.123)	0.276	0.917 (0.711-1.184)	0.508

Note: Adjusted for age, sex (female, male), ethnicity (whites, others), total energy intake (in tertiles, specified by sex), physical activity, smoking status, alcohol consumption, BMI (underweight/normal, overweight, obesity).

B

Crohn disease — Subgroup analysis (Mg quintiles: Q2-Q5 vs Q1)

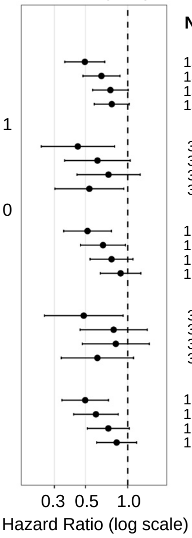

Subgroup	N (events)	HR (95% CI)	*p*
Overall			
Q5 vs Q1	150297 (401)	0.495 (0.356-0.686)	<0.001
Q4 vs Q1	150297 (401)	0.650 (0.480-0.881)	0.006
Q3 vs Q1	150297 (401)	0.753 (0.563-1.008)	0.056
Q2 vs Q1	150297 (401)	0.770 (0.578-1.025)	0.073
Medication use = 1			
Q5 vs Q1	34110 (118)	0.441 (0.242-0.807)	0.008
Q4 vs Q1	34110 (118)	0.609 (0.355-1.043)	0.071
Q3 vs Q1	34110 (118)	0.730 (0.435-1.227)	0.235
Q2 vs Q1	34110 (118)	0.533 (0.303-0.938)	0.029
Medication use = 0			
Q5 vs Q1	116187 (283)	0.517 (0.350-0.765)	<0.001
Q4 vs Q1	116187 (283)	0.667 (0.461-0.963)	0.031
Q3 vs Q1	116187 (283)	0.767 (0.539-1.090)	0.139
Q2 vs Q1	116187 (283)	0.890 (0.638-1.241)	0.492
Comorbidity = 1			
Q5 vs Q1	37860 (109)	0.486 (0.255-0.928)	0.029
Q4 vs Q1	37860 (109)	0.794 (0.458-1.378)	0.412
Q3 vs Q1	37860 (109)	0.823 (0.475-1.425)	0.486
Q2 vs Q1	37860 (109)	0.608 (0.336-1.100)	0.100
Comorbidity = 0			
Q5 vs Q1	112437 (292)	0.498 (0.340-0.729)	<0.001
Q4 vs Q1	112437 (292)	0.594 (0.412-0.856)	0.005
Q3 vs Q1	112437 (292)	0.728 (0.516-1.026)	0.070
Q2 vs Q1	112437 (292)	0.834 (0.601-1.157)	0.278

Ulcerative colitis — Subgroup analysis (Mg quintiles: Q2-Q5 vs Q1)

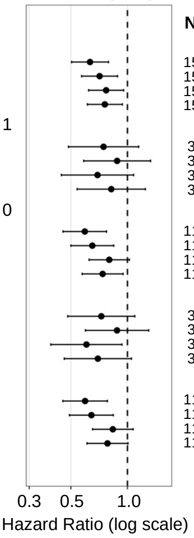

Subgroup	N (events)	HR (95% CI)	*p*
Overall			
Q5 vs Q1	150297 (763)	0.632 (0.504-0.793)	<0.001
Q4 vs Q1	150297 (763)	0.711 (0.570-0.886)	0.002
Q3 vs Q1	150297 (763)	0.770 (0.621-0.955)	0.017
Q2 vs Q1	150297 (763)	0.758 (0.612-0.940)	0.011
Medication use = 1			
Q5 vs Q1	34110 (208)	0.746 (0.485-1.148)	0.183
Q4 vs Q1	34110 (208)	0.881 (0.585-1.327)	0.545
Q3 vs Q1	34110 (208)	0.693 (0.445-1.079)	0.104
Q2 vs Q1	34110 (208)	0.820 (0.540-1.246)	0.353
Medication use = 0			
Q5 vs Q1	116187 (555)	0.593 (0.454-0.774)	<0.001
Q4 vs Q1	116187 (555)	0.650 (0.500-0.845)	0.001
Q3 vs Q1	116187 (555)	0.800 (0.625-1.024)	0.076
Q2 vs Q1	116187 (555)	0.738 (0.574-0.948)	0.017
Comorbidity = 1			
Q5 vs Q1	37860 (219)	0.726(0.481-1.095)	0.127
Q4 vs Q1	37860 (219)	0.880(0.597-1.299)	0.521
Q3 vs Q1	37860 (219)	0.605(0.391-0.935)	0.024
Q2 vs Q1	37860 (219)	0.696(0.461-1.051)	0.085
Comorbidity = 0			
Q5 vs Q1	112437 (544)	0.595(0.453-0.782)	<0.001
Q4 vs Q1	112437 (544)	0.643(0.491-0.841)	0.001
Q3 vs Q1	112437 (544)	0.836(0.652-1.073)	0.160
Q2 vs Q1	112437 (544)	0.783(0.609-1.007)	0.056

Irritable bowel syndrome — Subgroup analysis (Mg quintiles: Q2-Q5 vs Q1)

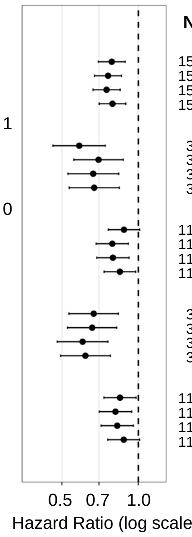

Subgroup	N (events)	HR (95% CI)	*p*
Overall			
Q5 vs Q1	150297 (2549)	0.786 (0.696-0.888)	<0.001
Q4 vs Q1	150297 (2549)	0.760 (0.672-0.859)	<0.001
Q3 vs Q1	150297 (2549)	0.750 (0.663-0.848)	<0.001
Q2 vs Q1	150297 (2549)	0.791 (0.701-0.893)	<0.001
Medication use = 1			
Q5 vs Q1	34110 (695)	0.585 (0.461-0.741)	<0.001
Q4 vs Q1	34110 (695)	0.697 (0.557-0.873)	0.002
Q3 vs Q1	34110 (695)	0.664 (0.528-0.837)	<0.001
Q2 vs Q1	34110 (695)	0.670 (0.534-0.842)	<0.001
Medication use = 0			
Q5 vs Q1	116187 (1854)	0.878 (0.762-1.013)	0.075
Q4 vs Q1	116187 (1854)	0.790 (0.682-0.914)	0.002
Q3 vs Q1	116187 (1854)	0.794 (0.686-0.919)	0.002
Q2 vs Q1	116187 (1854)	0.846 (0.733-0.977)	0.023
Comorbidity = 1			
Q5 vs Q1	37860 (715)	0.667 (0.533-0.834)	<0.001
Q4 vs Q1	37860 (715)	0.658 (0.526-0.822)	<0.001
Q3 vs Q1	37860 (715)	0.603 (0.479-0.759)	<0.001
Q2 vs Q1	37860 (715)	0.619 (0.494-0.777)	<0.001
Comorbidity = 0			
Q5 vs Q1	112437 (1834)	0.847 (0.732-0.980)	0.025
Q4 vs Q1	112437 (1834)	0.813 (0.702-0.942)	0.006
Q3 vs Q1	112437 (1834)	0.826 (0.714-0.956)	0.011
Q2 vs Q1	112437 (1834)	0.876 (0.758-1.012)	0.073

Diverticular disease — Subgroup analysis (Mg quintiles: Q2-Q5 vs Q1)

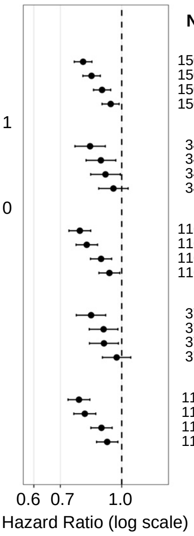

Subgroup	N (events)	HR (95% CI)	*p*
Overall			
Q5 vs Q1	150297 (15429)	0.799 (0.759-0.841)	<0.001
Q4 vs Q1	150297 (15429)	0.839 (0.798-0.883)	<0.001
Q3 vs Q1	150297 (15429)	0.892 (0.849-0.938)	<0.001
Q2 vs Q1	150297 (15429)	0.938 (0.892-0.985)	0.010
Medication use = 1			
Q5 vs Q1	34110 (5239)	0.832 (0.762-0.909)	<0.001
Q4 vs Q1	34110 (5239)	0.886 (0.813-0.966)	0.006
Q3 vs Q1	34110 (5239)	0.910 (0.835-0.992)	0.032
Q2 vs Q1	34110 (5239)	0.953 (0.875-1.038)	0.268
Medication use = 0			
Q5 vs Q1	116187 (10190)	0.784 (0.736-0.835)	<0.001
Q4 vs Q1	116187 (10190)	0.816 (0.767-0.869)	<0.001
Q3 vs Q1	116187 (10190)	0.887 (0.834-0.943)	<0.001
Q2 vs Q1	116187 (10190)	0.931 (0.876-0.989)	0.020
Comorbidity = 1			
Q5 vs Q1	37860 (5548)	0.837 (0.768-0.911)	<0.001
Q4 vs Q1	37860 (5548)	0.900 (0.828-0.978)	0.013
Q3 vs Q1	37860 (5548)	0.902 (0.829-0.981)	0.016
Q2 vs Q1	37860 (5548)	0.971 (0.894-1.054)	0.479
Comorbidity = 0			
Q5 vs Q1	112437 (9881)	0.780 (0.732-0.831)	<0.001
Q4 vs Q1	112437 (9881)	0.807 (0.757-0.860)	<0.001
Q3 vs Q1	112437 (9881)	0.889 (0.836-0.946)	<0.001
Q2 vs Q1	112437 (9881)	0.919 (0.864-0.978)	0.008

Celiac disease — Subgroup analysis (Mg quintiles: Q2-Q5 vs Q1)

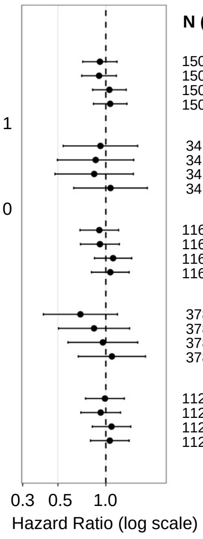

Subgroup	N (events)	HR (95% CI)	*p*
Overall			
Q5 vs Q1	150297 (669)	0.918 (0.716-1.176)	0.496
Q4 vs Q1	150297 (669)	0.907 (0.706-1.165)	0.444
Q3 vs Q1	150297 (669)	1.053 (0.826-1.343)	0.677
Q2 vs Q1	150297 (669)	1.065 (0.836-1.358)	0.608
Medication use = 1			
Q5 vs Q1	34110 (133)	0.925 (0.539-1.585)	0.776
Q4 vs Q1	34110 (133)	0.863 (0.496-1.499)	0.601
Q3 vs Q1	34110 (133)	0.845 (0.479-1.491)	0.561
Q2 vs Q1	34110 (133)	1.070 (0.628-1.824)	0.803
Medication use = 0			
Q5 vs Q1	116187 (536)	0.910 (0.688-1.203)	0.507
Q4 vs Q1	116187 (536)	0.918 (0.693-1.216)	0.552
Q3 vs Q1	116187 (536)	1.111 (0.848-1.455)	0.446
Q2 vs Q1	116187 (536)	1.067 (0.812-1.401)	0.642
Comorbidity = 1			
Q5 vs Q1	37860 (151)	0.692 (0.405-1.183)	0.178
Q4 vs Q1	37860 (151)	0.843 (0.504-1.411)	0.515
Q3 vs Q1	37860 (151)	0.958 (0.578-1.587)	0.868
Q2 vs Q1	37860 (151)	1.092 (0.671-1.777)	0.723
Comorbidity = 0			
Q5 vs Q1	112437 (518)	0.987 (0.745-1.306)	0.925
Q4 vs Q1	112437 (518)	0.928 (0.697-1.237)	0.612
Q3 vs Q1	112437 (518)	1.086 (0.822-1.434)	0.561
Q2 vs Q1	112437 (518)	1.059 (0.801-1.401)	0.686

FIGURE S7 Stratified and sensitivity analyses of dietary Mg intake and gut disorder risk.
(A) Sensitivity of Analysis of the associations between daily dietary Mg intake and the risk of five common gut disorders during the 13-year follow-up in the UK Biobank after excluding the under-nutrition subjects. (B) Stratified analyses by medication use and comorbidity status of the association between magnesium intake and gut disorders risk. Raw and processed data for drawing are provided in Source Data Figure 6.

Figure S8

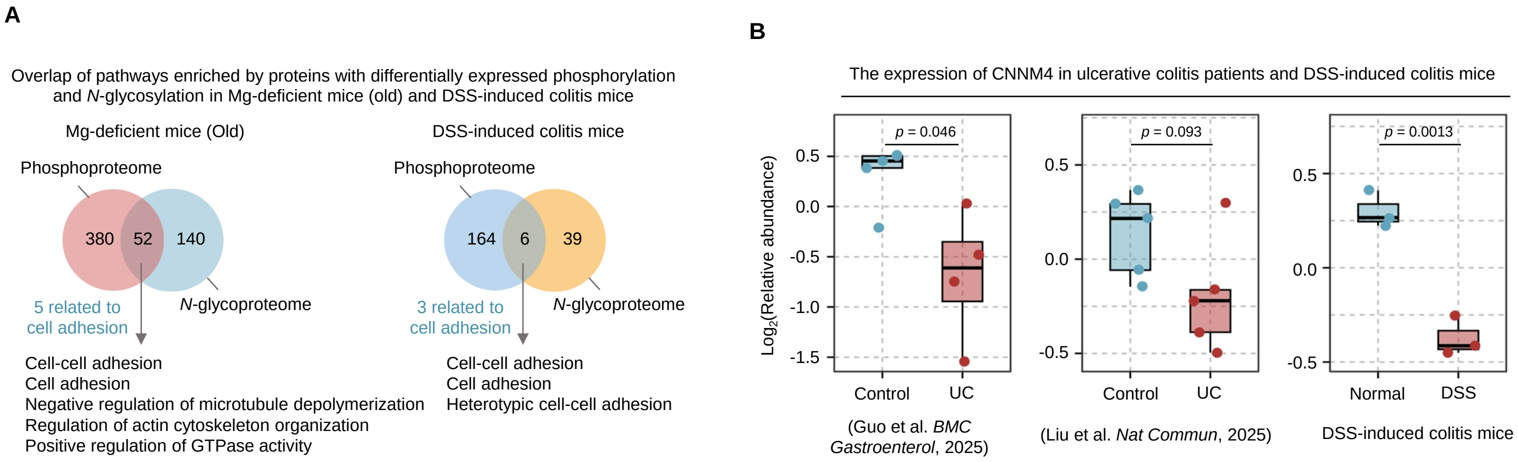

FIGURE S8 Comparisons between pathways involved in Mg-regulated phosphorylation and *N*-glycosylation.

(A) Venn diagram showing overlapping pathways enriched by proteins with differentially expressed phosphosites and *N*-glycopeptides in Mg-deficient mice (Old) and DSS-induced colitis mice. (B) Box plot displaying the levels of CNNM4 in ulcerative colitis patients and DSS-induced colitis mice (two-sided Student's *t*-test, median ± quartiles). Raw and processed data for drawing are provided in Source Data Figure S7. UC, ulcerative colitis.
